# Settling Velocities of Small Microplastic Fragments and Fibers

**DOI:** 10.1021/acs.est.3c09602

**Published:** 2024-03-21

**Authors:** Stefan Dittmar, Aki S. Ruhl, Korinna Altmann, Martin Jekel

**Affiliations:** † Chair of Water Quality Control, Technische Universität Berlin, Sekr. KF4, Straße des 17. Juni 135, 10623 Berlin, Germany; ‡ GEOMAR Helmholtz Centre for Ocean Research Kiel, Wischhofstraße 1−3, 24148 Kiel, Germany; § German Environment Agency (UBA), Section II 3.3, Schichauweg 58, 12307 Berlin, Germany; ∥ Bundesanstalt für Materialforschung und -prüfung (BAM), Unter den Eichen 87, 12205 Berlin, Germany

**Keywords:** microplastics, fragments, fibers, transport, settling velocity, sinking velocity, sedimentation

## Abstract

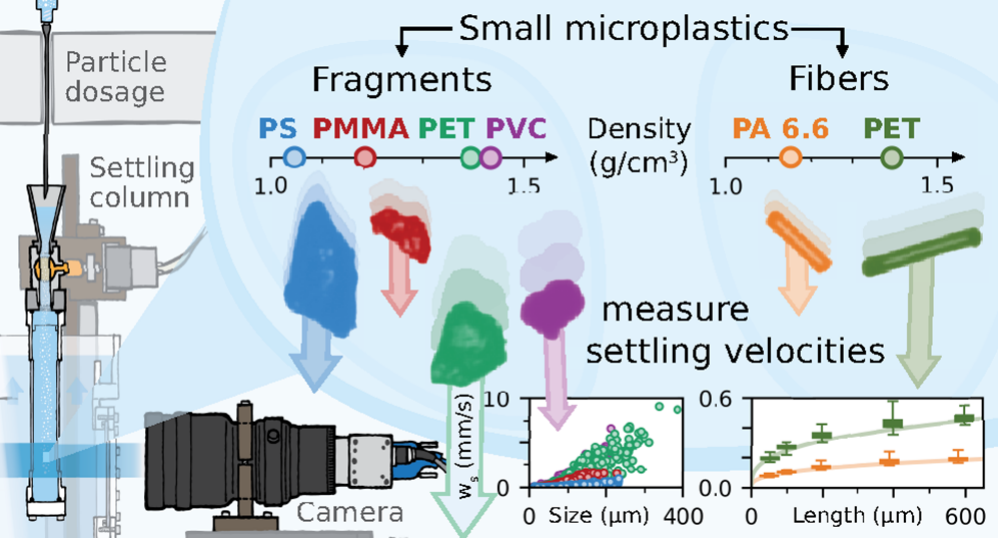

There is only sparse empirical data on the settling velocity of small, nonbuoyant microplastics thus far, although it is an important parameter governing their vertical transport within aquatic environments. This study reports the settling velocities of 4031 exemplary microplastic particles. Focusing on the environmentally most prevalent particle shapes, irregular microplastic fragments of four different polymer types (9–289 μm) and five discrete length fractions (50–600 μm) of common nylon and polyester fibers are investigated, respectively. All settling experiments are carried out in quiescent water by using a specialized optical imaging setup. The method has been previously validated in order to minimize disruptive factors, e.g., thermal convection or particle interactions, and thus enable the precise measurements of the velocities of individual microplastic particles (0.003–9.094 mm/s). Based on the obtained data, ten existing models for predicting a particle’s terminal settling velocity are assessed. It is concluded that models, which were specifically deduced from empirical data on larger microplastics, fail to provide accurate predictions for small microplastics. Instead, a different approach is highlighted as a viable option for computing settling velocities across the microplastics continuum in terms of size, density, and shape.

## Abstract 2

This study reports measurements of small microplastic fragments (9−289 μm) and fibers (50−600 μm length) settling in quiescent water and assesses the applicability of existing models for computing terminal settling velocities.

## Introduction

Microplastics (MPs) have become ubiquitous in the environment and are found even in the remotest habitats.^1−3^ MPs are briefly defined as solid particles mainly consisting of polymers between 1 μm and 1 mm in size, while particles between 1 and 5 mm are referred to as “large MPs”.^4^ The definition thus encompasses a wide range of particles that differ not only in size but also in their chemical composition, surface properties, density, and shapes.^5,6^ Size, shape, and density of each individual particle determine its settling velocity in a quiescent fluid of lower density.^7^ Within aquatic environments, the settling velocity of a nonbuoyant MP particle is one of the most important factors, influencing its vertical transport and fate, alongside flow characteristics, resuspension, and possible interactions with other particles and biota,^8^ such as heteroaggregation,^9−11^ biofilm formation,^12,13^ and ingestion.^14,15^ MPs’ settling velocities are not only important input parameters for transport models^16−18^ but should be also considered with respect to strategies and methods of environmental sampling.^19,20^

According to particle numbers, the predominant MPs found in marine and freshwater ecosystems are irregularly shaped fragments smaller than 500 μm and fibers with lengths below 1 mm,^21−25^ although cellulose and semisynthetic fibers are sometimes probably misidentified as MPs as well.^26,27^ While many studies reported empirical settling velocities of larger MPs,^28−40^ only few investigated the settling of these smaller particles yet, despite their prevalence and a supposedly higher ecotoxicological relevance.^41^ Certain flow conditions might reduce the significance of small MPs’ settling velocities,^19^ yet it was concluded by Hoellein et al.^42^ that nominal settling velocities of small MPs could provide good estimations of their actual deposition velocity in natural streams.

Kaiser et al.^43^ measured settling velocities of MP fragments between 6 and 256 μm. Nguyen et al.^44^ observed the settling of polyurethane (PUR) fragments (50–500 μm) and bigger, microbial-associated PUR aggregates. Other studies^45−47^ include limited numbers of fragments smaller than 500 μm but mainly focus on larger MPs as they rely on the handling of individual particles. Regarding MP fibers, settling velocities have only been measured for lengths above 1 mm so far,^30,33,34,37,38,47^ except for data on fishing line cuts down to a length of 500 μm,^31^ whose diameters (150–710 μm) exceed those of fibers usually found in environmental samples (5–50 μm).^25,27^

This study presents settling velocities of typical small, pristine and nonbuoyant MPs measured in quiescent water using a high-precision measurement setup that was exclusively designed for this purpose and has been validated extensively by Dittmar et al.^48^ MP fragments (9–289 μm) of four polymer types, polystyrene (PS), poly(methyl methacrylate) (PMMA), poly(ethylene terephthalate) (PET), and poly(vinyl chloride) (PVC), were investigated alongside polyamide 6.6 (PA 6.6) and PET fibers, which were cut to five different length fractions (50, 100, 200, 400, and 600 μm), respectively.

Most challenges in investigating MPs arise from the heterogeneity of the considered particle population. Therefore, Kooi et al.^5^ proposed to conceptualize MPs by means of continuous distributions of particle size, density, and shape, which is more and more adopted in current research.^49,50^ Transferring this approach to computing the settling and rising velocities of MPs consequently favors the use of universal formulas, which can be continuously applied to the entire MP spectrum. In conclusion, the measurement results for small MPs obtained in this study are used to assess the predictive qualities of ten different settling velocity models, six of which were specifically reported for MPs.

## Materials and Methods

### Particle Samples

PVC fragments and corresponding PVC pellets (Granulate 6610) were kindly provided by Vestolit GmbH (Germany). The remaining MP fragments were produced by milling pristine polymer pellets: PS (Polystyrol 158 K, BASF, Germany) and PET (Lighter C93, Equipolymers, Netherlands) pellets were precooled in liquid nitrogen for 3 min, transferred to a centrifugal mill (ZM200, Retsch, Germany) equipped with a stainless steel ring sieve with holes of 500 μm, and then milled at 16,000 rpm. PMMA pellets were provided by PlasticsEurope (Germany) and cryo-milled in a ball mill (Cryomill, Retsch, Germany): Approximately 2 g of pellets were filled into a milling jar (25 mL) with five stainless steel balls of 12 mm diameter, then precooled for 5 min at 5 Hz, and ground over 6 cycles of milling (5 min at 25 Hz) and cooling (30 s at 5 Hz). The milling jar was cooled with liquid nitrogen throughout. All obtained powders were sieved (<500 μm) to remove any larger MP fragments. Pellet densities were determined in triplicate at ambient conditions (20 °C) via displacement of ethanol using a pycnometer (50 mL, Marienfeld, Germany) and consequently assumed as densities of the respective fragments: 1.046 ± 0.001 g/cm^3^ for PS, 1.187 ± 0.003 g/cm^3^ for PMMA, 1.396 ± 0.003 g/cm^3^ for PET, and 1.435 ± 0.001 g/cm^3^ for PVC.

Following the protocol of Cole,^51^ PA 6.6 and PET fibers with a nominal diameter of 23 μm (Goodfellow, U.K.) were embedded in a water-soluble cryo-embedding compound (OCT Embedding Matrix, CellPath, U.K.) and cut with a cryogenic microtome (CM1950, Leica Biosystems, Germany) to produce MP fibers at the desired lengths of 50, 100, 200, 400, and 600 μm. These fractions were chosen since they cover the range of fiber lengths predominantly found in environmental samples.^22,25^ PA 6.6 and PET were selected to represent polyamides and polyesters, respectively, which are predominant groups of polymers used in the manufacture of synthetic textiles.^52,53^ Detailed methodical instructions can be found in a Zenodo repository,^54^ including slight modifications and improvements of the original protocol^51^ with regard to fiber embedding. Fiber diameters and mean lengths of each fraction were determined from microscopic images (Axioskop, Zeiss, Germany). Respective fibers were recovered from the settling column (cf. “Settling Experiments” section) via vacuum filtration on a polycarbonate membrane (8 μm pore size, Millipore) after completing the settling experiments and then dried at 35 °C. The fibers were resuspended in ultrapure water (ELGA Berkefeld LabWater, resistivity >17 MΩ/cm) 48 h prior to image acquisition in order to account for water absorption of PA 6.6.^55^ The analysis of acquired microscopy images was performed manually, facilitated by a self-developed Python user interface. Between 193 and 561 fiber lengths were measured per fraction. The complete data is included in the Zenodo repository.^54^ Densities of PA 6.6 (1.152 ± 0.004 g/cm^3^) and PET fibers (1.393 ± 0.004 g/cm^3^) were determined by observing visible cuts suspended in graded density solutions of NaCl or ZnCl_2_, respectively. The method has been previously described for PS spheres.^48^ Due to low settling velocities of the fibers, the suspensions were left to settle for 12 h instead of 1 h.

**Figure 1:**
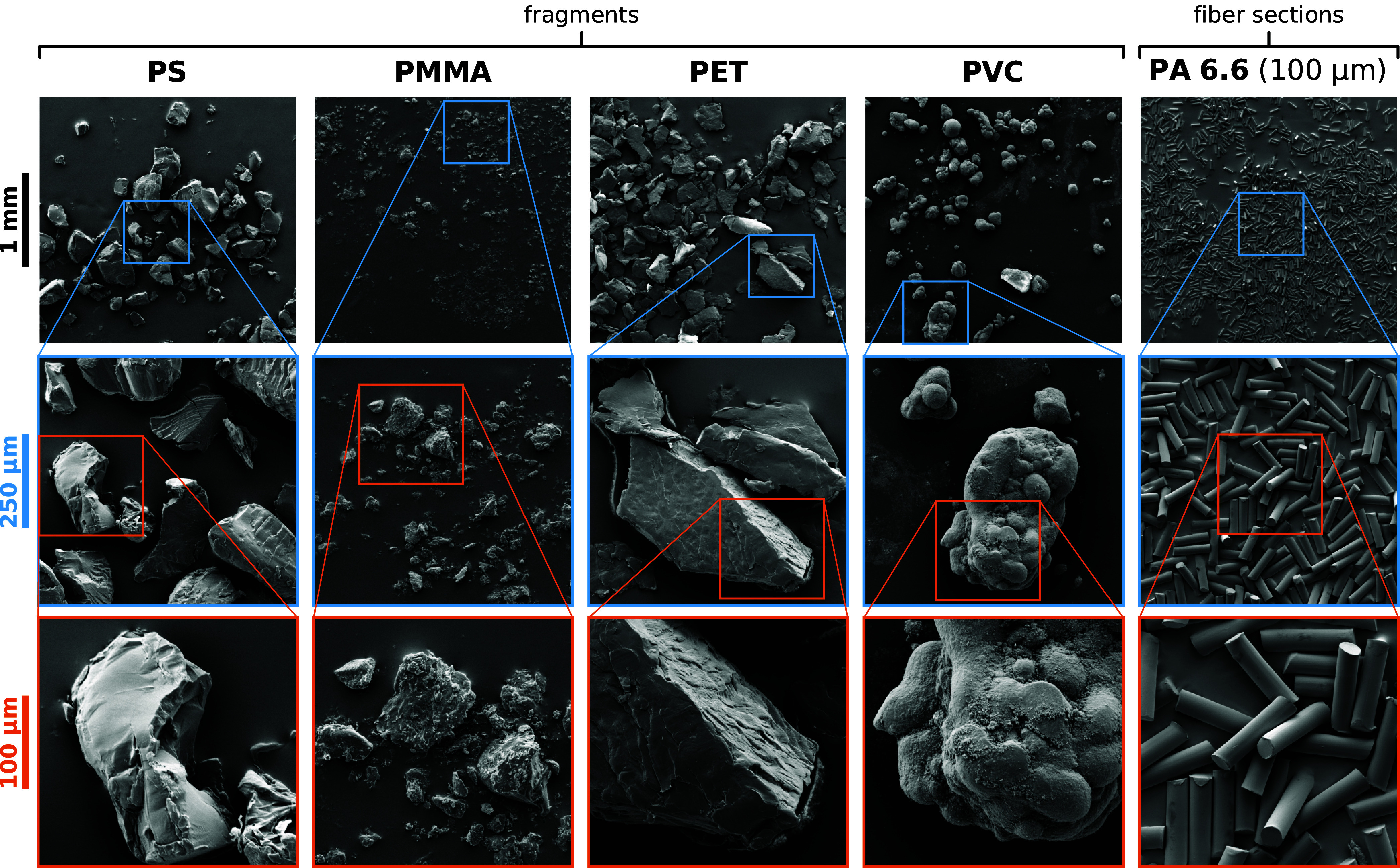
Exemplary scanning electron microscopy (SEM) images of all investigated MP fragments and the 100 μm length fraction of PA 6.6 fibers (columns). Three uniform magnifications are displayed (rows, note color codes, and scale bars on the left-hand side), and the magnified regions are indicated, respectively. All samples were Au sputtered before SEM images were acquired in secondary electron mode.

Figure 1 shows images of all MP fragments and an exemplary length fraction of PA 6.6 fibers taken with a scanning electron microscope (Gemini DSM 982, Zeiss, Germany). Complete imagery is provided in the Supporting Information (Section S1 and Figures S1–S3).

### Settling Experiments

Settling velocities of MPs were measured via optical imaging using the setup and method that was described and validated by Dittmar et al.^48^ High measurement accuracy is achieved by effectively suppressing thermally induced convection flows. Moreover, an empirical model was proposed to monitor interactions between settling particles, which potentially alter the measured settling velocities.^48^ The model can aid in minimizing such particle–particle interactions by adjusting the respective particle dose (e.g., according to preliminary experiments). See the original publication^48^ for comprehensive details on the method and its validation. Additional information is given in the following: All experiments were carried out at 15 °C. MP particles were dosed from stock suspensions prepared with ultrapure water, which was previously spiked with the surfactant mix NovaChem SF100 (Postnova Analytics, Germany) and degassed for 15 min. SF100 was added in equal concentration to the targeted mass concentration of MPs in order to ensure particle stabilization. Thus, the formation of aggregates was prevented, despite elevated MP concentrations. Within aquatic environments, adsorption of natural organic matter^56^ as well as decreased surface hydrophobicity due to weathering^57^ can promote stabilization of MPs. Immediately before each dosage, stock suspensions were rehomogenized by shaking. The dosing volume (10 mL for MP fragments or 3.5 mL for MP fibers, respectively) was transferred using a syringe with a cannula (120 mm length, 2 mm inner diameter).

MP fragments were investigated in consecutive sets of experimental runs with a single, initial particle dose, always reducing runtime from set to set but in turn increasing the number of runs and mostly the particle dose, too. Thus, an increased number of larger fragments can be captured: They are usually less frequent, yet assumed to be less prone to particle–particle interactions that potentially alter their velocity.^48^ For sets with a reduced runtime, a matching minimum size is specified for a particle to be finally evaluated. Table 1 comprises these size cutoffs as well as particle doses, runtimes, and initial frame rates of all experiments with MP fragments.

**Table 1:** Overview of All Sets of Settling Experiments Conducted with the MP Fragments

sample name	set	runs	particle dose per run (mg)	runtime	initial frame rate (Hz)^a^	minimum particle size^b^
PS fragments	set 1	8	4.0	27 h	4	none
	set 2	30	4.0	1 h	4	80 μm

PMMA fragments	set 1	20	0.1	9 h	2	none
	set 2	20	0.4	1 h 30 min	2	40 μm
	set 3	20	1.6	18 min	2	80 μm

PET fragments	set 1	15	0.8	5 h	10	none
	set 2	40	0.8	45 min	10	50 μm
	set 3	80	1.6	16 min	10	100 μm

PVC fragments	set 1	40	0.2	2 h	10	none
	set 2	64	0.5	9 min	10	60 μm

a Frame rate dynamically reduced with increasing runtime according to Dittmar et al.^48^

b fixed minimum equivalent circular diameter (ECD) for a detected particle to be considered.

As MP fibers were investigated in separate length fractions, the settling experiments were conducted following the dosage scheme for monodisperse particles proposed by Dittmar et al.^48^ Suspension containing the target particles (5 mg/L) was dosed repeatedly at constant intervals, which resulted in nominal single doses of 17.5 μg. Dosing intervals were derived from preliminary experiments and are summarized in Table 2 for each fraction along with the respective frame rate and number of doses.

**Table 2:** Overview of All of the Settling Experiments with MP Fibers

polymer	length fraction (μm)	doses	dosing interval (min)	frame rate (Hz)
PA 6.6	50	20	20	0.15
	100	30	14	0.20
	200	40	10	0.25
	400	50	8	0.30
	600	60	7	0.40
PET	50	20	9	0.40
	100	30	7	0.40
	200	40	6	0.50
	400	50	5	0.60
	600	60	4	0.70

### Image Processing and Data Quality

All raw image sequences acquired during the settling experiments were processed with self-developed Python scripts for particle tracking. The scripts are publicly available.^58^ Their functionality and parameters are explained in a previous publication^48^ and its corresponding Supporting Information. Processing parameters were set in accordance with a detection accuracy of ±1 μm, referring to the equivalent circular diameter (ECD) of a detected particle contour.^48^ Particles were only considered, if they were detected in at least 4 frames and, in order to avoid duplicate detections, if they were tracked along at least half the field of view’s (FOV) vertical extent minus the maximum height of the particle contour. For processing data on fibers, the parameter similarity threshold was increased from 2 to 4 in order to account for a potential rotation of the particles and their large aspect ratio (cf. Dittmar et al.^48^ and corresponding Supporting Information for details on the image processing parameters).

To ensure the integrity of settling measurements, detected contours of all tracked particles were visually inspected by one human observer, e.g., to identify obvious particle contaminations. Moreover, the empirical model for particle–particle interactions, proposed specifically for the employed experimental setup,^48^ was applied to contain particle clustering effects on the measured velocities: Tracked particles, whose modeled deviation from the single particle settling velocity exceeded 0.01 mm/s as well as 5% of the measured velocity, were excluded from evaluation. All results of these data filtering procedures are presented and discussed in Section S2 of the Supporting Information.

### Terminal Settling Velocity Models

Ten different existing models for predicting a particle’s terminal settling velocity are compared based on the empirical data acquired for small MP fragments and fibers. Six of those models were derived specifically for MPs (models M1–M6, as annotated in the following), while the remaining four models (models M7–M10) are of a more general nature and were selected from other fields. First of all, balancing gravitational, buoyancy, and drag force exerted on a particle settling in a quiescent fluid yields the following expression for its terminal settling velocity *w*_s_
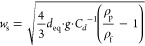
1with equivalent spherical diameter *d*_eq_, gravitational constant *g*, fluid density ρ_f_, particle density ρ_p_, and drag coefficient *C*_*d*_. *C*_*d*_ is a function of particle shape and surface friction as well as the flow properties comprised by the Reynolds number Re, which is determined by *w*_s_, the fluid’s kinematic viscosity ν, and a characteristic diameter *d* (e.g., *d*_eq_) as follows
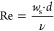
2

Since all settling experiments were conducted at 15 °C, respective fluid properties of pure water are assumed throughout (ρ_f_ = 0.999 g/cm^3^, ν = 1.140 × 10^–6^ m^2^/s). The terminal settling velocity can then be implicitly calculated by using eq 1 and a suitable expression for *C*_*d*_. Based on own empirical data of mainly large MPs, both Waldschläger and Schüttrumpf^47^ (M1) and Goral et al.^40^ (M2), respectively, proposed separate *C*_*d*_ for MP fragments or MP fibers. Using similar, compiled data sets, Yu et al.^59^ (M3) and Zhang and Choi^60^ (M4) each proposed a general *C*_*d*_ for MPs. So far, only Kaiser et al.^43^ (M5) specifically addressed small MPs, presenting an explicit, empirical equation for *w*_s_. A similar equation was proposed for larger MP fibers by Khatmullina and Isachenko^31^ (M6).

Apart from MP-specific approaches, *C*_*d*_ models derived by Bagheri and Bonadona^61^ (M7) and Dioguardi et al.^62^ (M8) are assessed. Moreover, Komar et al.^63^ (M9) described the *C*_*d*_ of ellipsoids and cylinders at laminar flow conditions, which is applied to MP fragments or MP fibers, respectively. Finally, an approach by Su et al.^64^ (M10) for computing settling velocities over all subcritical flow regimes (Re < 3 × 10^5^) and for various shapes, generalized as superellipsoidal particles, is evaluated. Details on the implementation of these models are given in Section S4 of the Supporting Information.

Most settling velocity models require the equivalent spherical diameter *d*_eq_ of a considered particle as the input. In contrast to studies investigating the settling of larger MPs,^33,40,47^ individual MP fragments can only be characterized based on their contours detected in raw images from settling experiments. Consequently, *d*_eq, MP fragment_ is computed following an appropriate correction formula of Bagheri et al.^65^
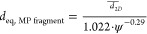
3 is calculated as the mean ECD of the three contours with minor, major, and nearest to average projection area, respectively. The sphericity ψ is approximated from the contour circularity. The computation is detailed in Section S3 of the Supporting Information, including further shape descriptors required for the implementation of the velocity models.

For each fraction of MP fibers, *d*_eq_ can be estimated from diameter *D* and mean length *L* assuming a cylindrical shape
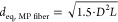
4

Following previous studies,^33,66^ the average absolute relative error (|AE|) was computed from all *N* pairs of measured and predicted settling velocities in order to assess the performance of a terminal settling velocity model
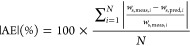
5Furthermore, coefficients of determination were calculated based on the absolute errors (*R*^2^) and the logarithms of the absolute errors (*R*_log_^2^), respectively, the latter in order to pronounce lower settling velocities, since the measuring range spans more than 3 orders of magnitude.

## Results and Discussion

### Microplastic Fragments

**Figure 2:**
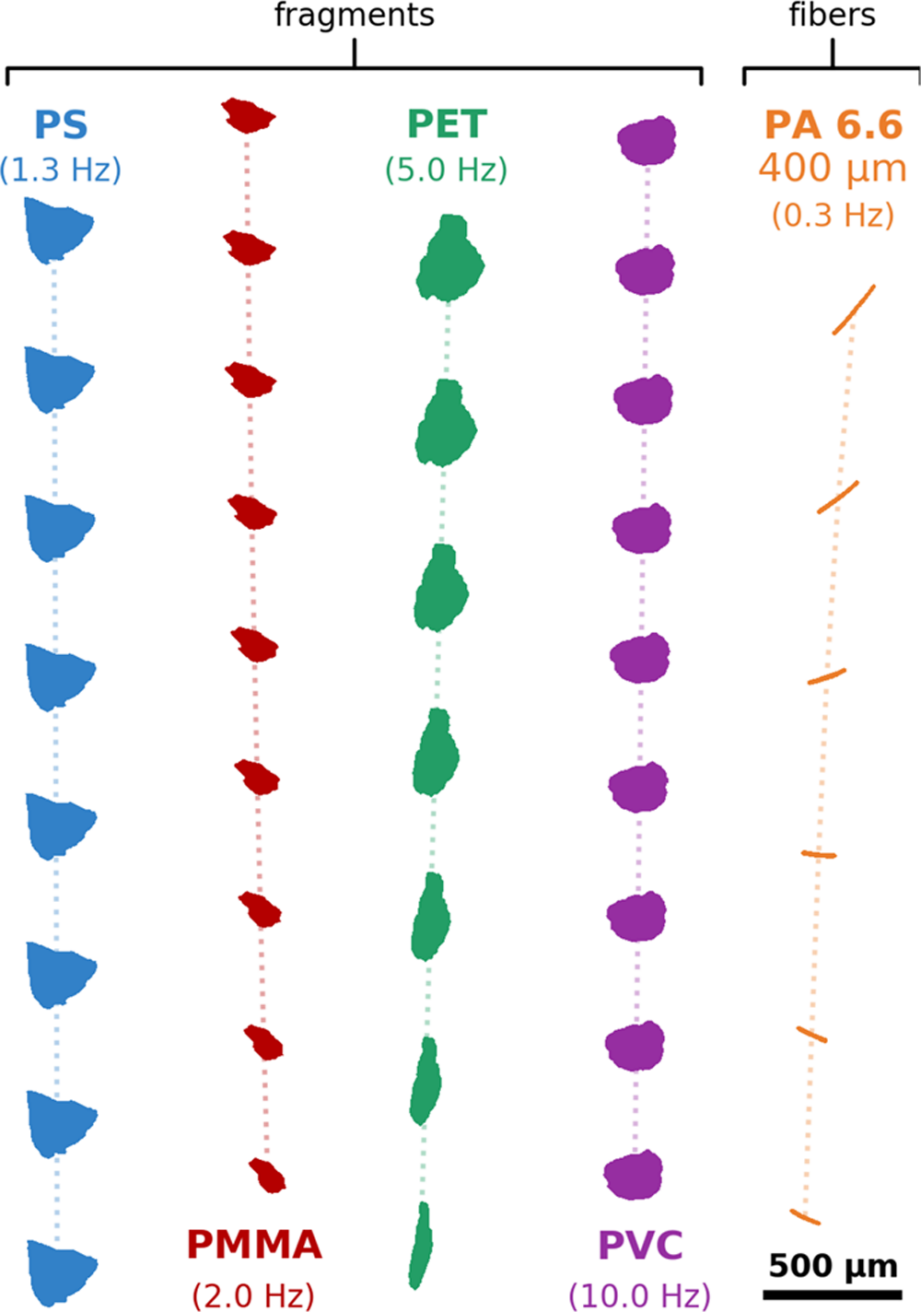
Exemplary trajectories of different MP fragments and one 400 μm long fiber obtained from settling experiments. Note the scale bar and the respective image acquisition frame rate (annotated in brackets).

The investigated nonbuoyant MP fragments did not only vary in terms of polymer type and thus density (1.046–1.435 g/cm^3^) but also regarding particle shape due to different milling techniques and material properties (e.g., brittleness and glass-transition temperature). From the SEM images (cf. Figure 1), PVC fragments appear rather rounded, while other fragments exhibit a more angular shape. Especially PET fragments appear as mostly flat, flake-like particles with straight edges and large areas of plane surfaces and deviate most strongly from a spherical shape. These observed differences are supported by data acquired from the settling experiments. Figure 2 shows the settling trajectories of exemplary MP fragments and one fiber.

**Figure 3:**
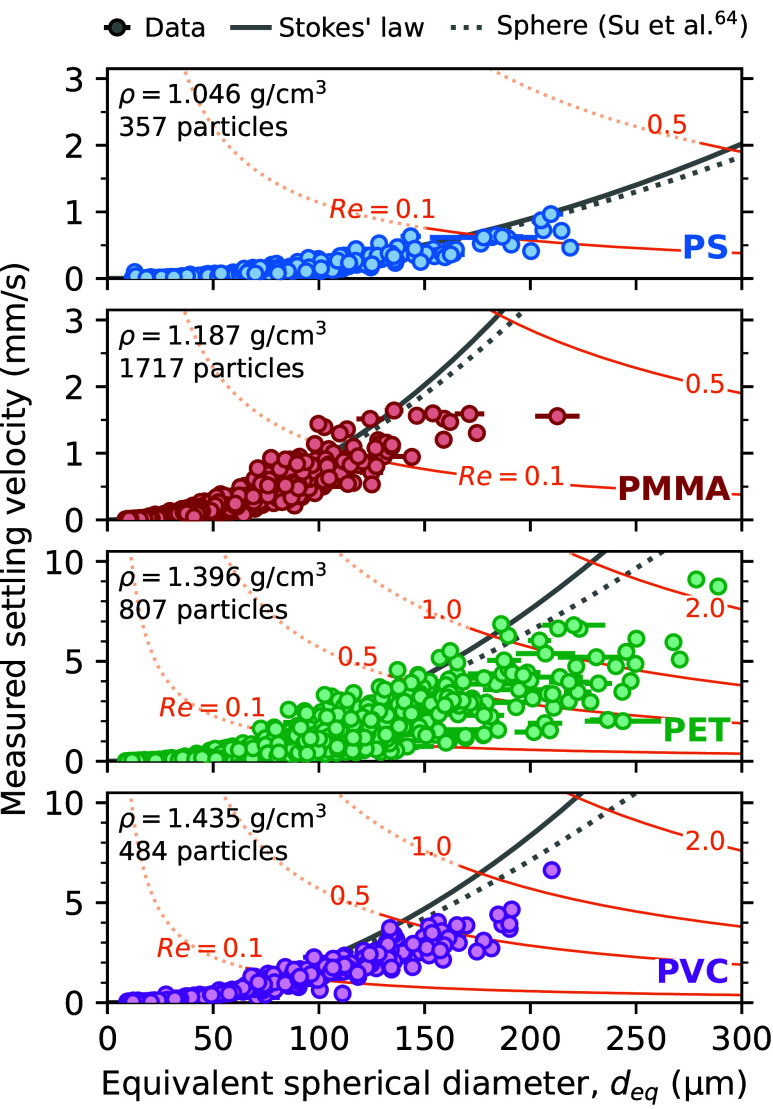
Measured settling velocities at 15 °C and equivalent spherical diameters of all of the investigated MP fragments. Note the variation of *y*-axis scaling for the different polymer types. Terminal settling velocities predicted for spherical particles of the respective densities ρ are plotted according to Stokes’ law (valid for laminar flow, Re ≪ 1) and the model of Su et al.^64^ Distinct Reynolds numbers are annotated (orange).

The PET fragment depicted in Figure 2 rotates vertically, and its flatness is revealed by the large variation of the contour areas. Consequently, the deduced equivalent diameter *d*_eq_ (164.2 ± 40.5 μm) shows a high relative standard deviation of 24.7%, whereas it amounts to 9.6, 0.6, and 0.4% for the depicted PMMA, PS, and PVC particles. When considering all measurements, a median relative standard deviation of *d*_eq_ of 6.5% and a median aspect ratio (cf. Table S1) of 2.47 were observed for PET fragments, compared to only 1.6% and 1.45 for PVC fragments (also see error bars for *d*_eq_ in Figure 3). This difference emphasizes the irregularity of PET fragments as well as the general difficulty of characterizing such particles’ size based on 2D imaging.^65^ Not every tracked particle rotates, which would allow for the capture of significantly different contour projections. Although addressed by specific corrections, e.g., eq 3 proposed by Bagheri et al.,^65^ this lack of information can only be partially compensated. To contain uncertainties across the whole size range of the investigated samples, settling experiments were structured with the aim of capturing an increased number of larger particles (cf. “Settling Experiments” and size cutoffs in Table 1).

In total, the settling velocities of 3365 MP fragments could be measured successfully. Figure 3 depicts the measurement results for each investigated polymer type of MP fragments. All underlying data are available from the Zenodo repository.^54^ The measured settling velocities and equivalent spherical diameters range from 0.003 to 9.094 mm/s and from 8.6 to 288.9 μm, respectively. The minimum particle size is indirectly imposed by the detection limit of 10 μm given for the ECD, which is then corrected to *d*_eq_ (cf. eq 3). The Reynolds numbers given in Figure 3 indicate that the majority of measurements can be attributed to the laminar flow regime (Re ≪ 1), with few exceptions regarding larger PET and PVC fragments. The scatter of measured velocities and corresponding particle diameters is notably higher for PET and PMMA compared to PVC fragments. As discussed above, this scatter can most likely be attributed to their irregular shapes and associated uncertainties of estimating particle sizes. For 13% of the tracked MP fragments, the measured velocities even exceed the terminal settling velocity predicted for a sphere of equal estimated volume. The computed velocities of settling spheres are plotted in Figure 3 according to Stokes’ law (Re ≪ 1) and the model of Su et al.,^64^ which extends further to the transitional regime, in order to provide optical guidance in the form of reasonable upper estimates for settling velocity. Additionally, the drag coefficient *C*_*d*_ was calculated from the observed settling velocity *w*_s_ and equivalent diameter *d*_eq_ for each measured MP fragment and each MP fiber. Respective results are presented in the Supporting Information (Section S6), e.g., by plotting *C*_*d*_ values versus Reynolds numbers (cf. Figure S16). The full underlying data is provided in the Zenodo repository.^54^

In general, terminal settling velocities were measured with very high precision, e.g., the velocities measured between consecutive detections of one particle showed an average standard deviation of only 0.001 mm/s.

### Microplastic Fibers

**Figure 4:**
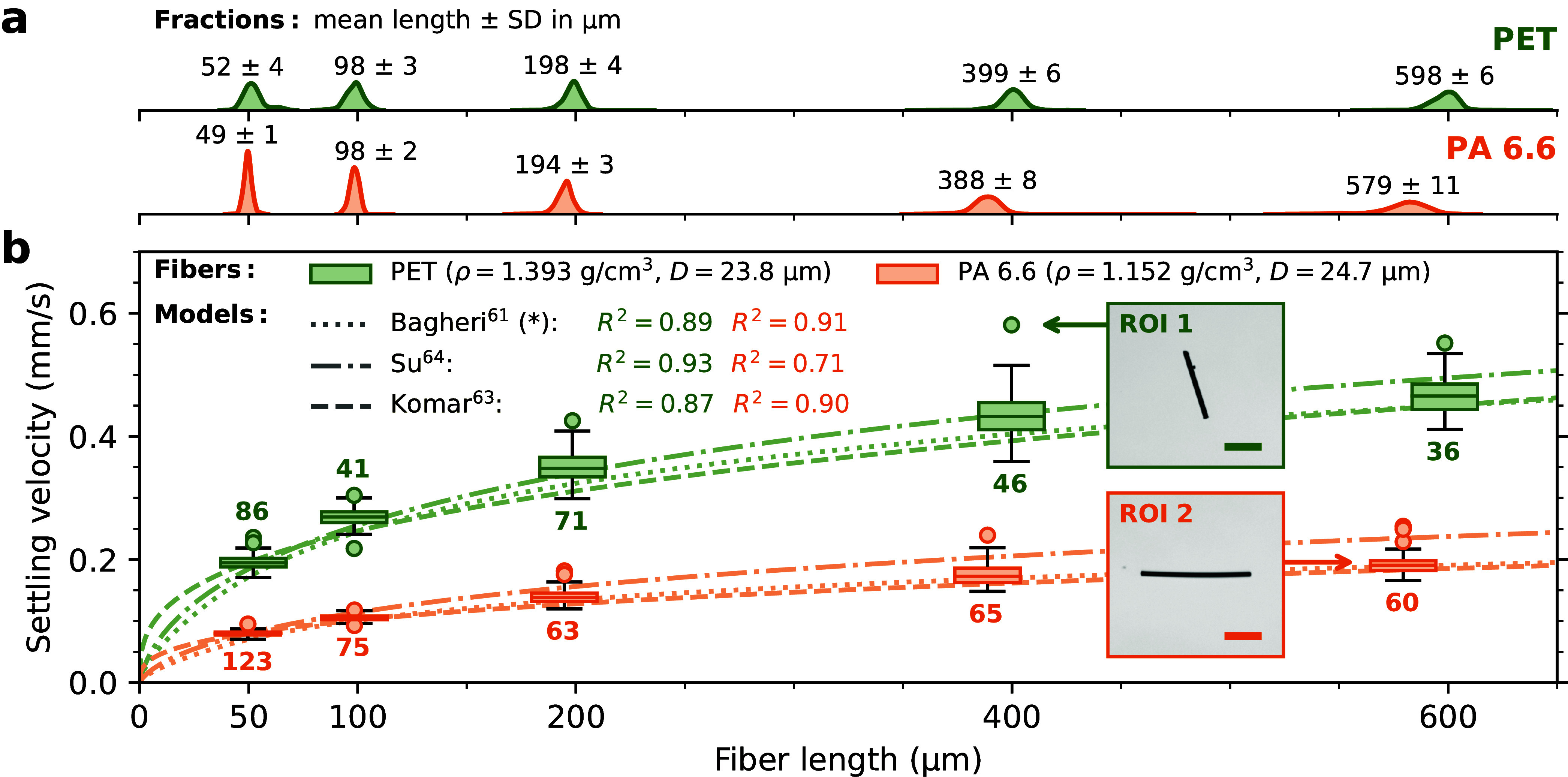
Length distributions (a) and boxplots of settling velocities measured at 15 °C (b) of all investigated fractions of PET and PA 6.6 fibers. Mean lengths and standard deviations (cf. a) as well as the number of fibers per fraction measured during settling experiments (cf. b) are annotated next to the respective plots. The best tested terminal velocity models are plotted with coefficients of determination *R*^2^ given for PA 6.6 and PET in respective colors (cf. Section S7 of the Supporting Information; (*) indicates the use of *d*_2_ instead of *d*_eq_ as input diameter). Two regions of interest (ROIs) of raw images are inset to highlight fibers settling with different orientations: The arrows indicate their respective velocities, and colored scale bars denote 200 μm.

It is difficult to determine the length of settling fibers based on 2D imaging. Due to their high aspect ratio (up to 25 in this study), their apparent length depends on the orientation toward the image plane (cf. exemplary trajectory in Figure 2). In order to avoid this ambiguity, discrete length fractions were produced from both PA 6.6 and PET fibers and were investigated in separate settling experiments. In total, 666 MP fibers were captured successfully. Figure 4 shows measured settling velocities and length distributions for each fraction. Lengths and diameters were determined microscopically. The diameters of wetted PET and PA 6.6 fibers are 23.8 and 24.7 μm, respectively. As PA 6.6 is hygroscopic, the diameter increases by 1.0 μm compared to dry fibers. This increase corresponds to 8 vol % of absorbed water, which is in accordance with the literature.^55^ The 200, 400, and 600 μm fractions of PA 6.6 fall slightly below their nominal length. Nevertheless, all fractions show high length uniformity with relative standard deviations below 4.1%, with the exception of the 50 μm fraction of PET (8.7%). Consequently, the mean measured length is assumed throughout for all particles of one fraction.

Recorded settling velocities ranged from 0.070 to 0.254 mm/s for PA 6.6 and from 0.171 to 0.581 mm/s for PET fibers. On average, the velocities increase with fiber length, yet the rate of increase is clearly declining. When comparing the 400 and 600 μm length fractions, the average velocity increases by only 9.3% for PA 6.6 and 7.7% for PET. Most fibers were aligned horizontally to the settling direction (cf. ROI 2 in Figure 4): 72.2% deviate less than 10° from horizontal orientation, which further increases to 82.1% if only length fractions between 200 and 600 μm are considered (computation detailed in Section S5 of the Supporting Information). Both observations are generally in line with previous studies on the settling of larger MP fibers.^37,38,47^ Nguyen et al.^37^ also noted that vertically aligned fibers (>1 mm) settle up to 1.7 times as fast as horizontally aligned fibers due to decreased drag. A similar velocity increase by 33% was highlighted for a 400 μm PET fiber (see ROI 1 in Figure 4). Unlike longer MP fibers,^30,37^ the fibers investigated in this study showed no considerable curliness or curvature (cf. ROIs in Figure 4). On average, the settling fibers’ orientation varied by ±0.8° between frames and their apparent length, considered relatively, by only ±3.2%. These average values are far less than the dramatic example shown in Figure 2, which had ±8.1° variation in orientation and ±23.7% variation in length. The low average values thus indicate only minimal secondary motions^38^ for the majority of the measured MP fibers.

To provide further guidance, Figure 4 also includes predicted terminal settling velocities according to the three best-performing models that were tested in this study. The predictions are plotted for both PA 6.6 and PET fibers of respective diameter and density across the considered length range. The models of Bagheri and Bonadonna^61^ and Komar^63^ are in good accordance with the measurement data on MP fibers, achieving coefficients of determination (*R*^2^) between 0.87 and 0.91 as well as average absolute relative errors (|AE|) between 6.1 and 8.6% for PA 6.6 and PET (cf. Table S4). Both models’ predictions improve, when evaluating only fibers that deviate less than 10° from horizontal alignment (*R*^2^: 0.88–0.94, |AE|: 5.3–7.8%), since none of the considered models accounts for varying orientation of settling particles. All tested models are discussed in detail in the next section.

### Applicability of Terminal Settling Velocity Models

**Figure 5:**
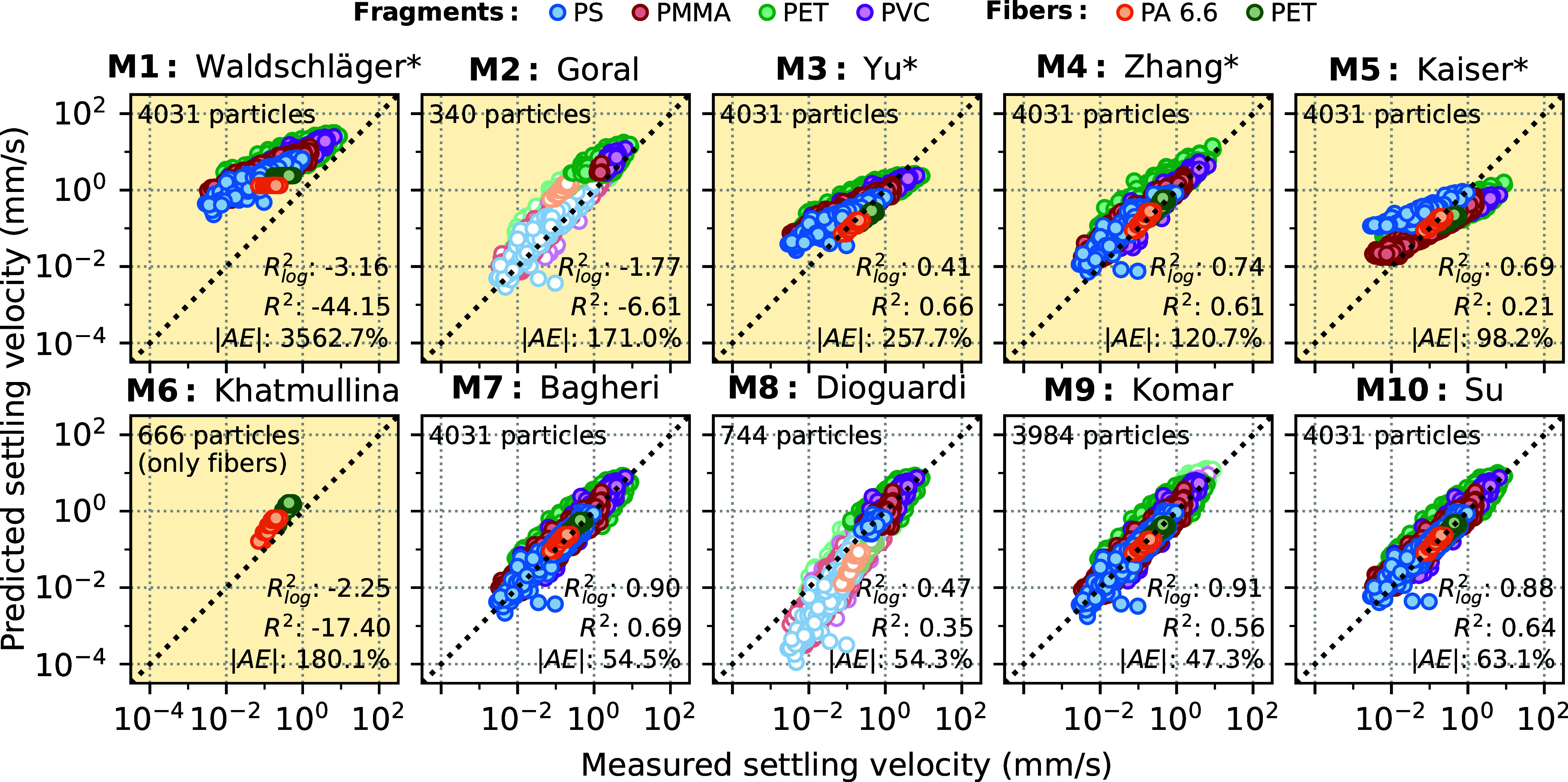
Measured settling velocities versus corresponding predictions by each of the tested terminal settling velocity models (note the log–log scale). Performance measures *R*_log_^2^, *R*^2^ and |AE| are indicated, respectively. If a validity range is specified for a model according to the Reynolds number, outliers are not considered and are depicted pale. Use of *d*_2_ instead of *d*_eq_ as input diameter is indicated by * (see Section S7). Models derived specifically for MP are highlighted with a yellow background.

Figure 5 depicts measured velocities for all 4031 investigated MP fragments and fibers as well as corresponding predictions by each of the ten different models for computing terminal settling velocities, which were assessed. If the respective model specifies requirements regarding particle shape or Reynolds number, only appropriate data are considered for computing the depicted performance measures: Coefficients of determination calculated from absolute (*R*^2^) or logarithmic errors (*R*_log_^2^) as well as the average absolute relative error |AE| (cf. eq 5). Besides *d*_eq_ (see eqs 3 and 4), three alternative definitions for determining the equivalent diameter were tested in order to account for possible ambiguities when parametrizing particle size. The Supporting Information includes further details (Section S7) and full results (Figures S17–S19). Tables S2–S4 comprise the assessments of the predictive qualities of each tested model by means of all three performance measures: differentiating between the full data set, MP fibers, MP fragments, and each investigated particle type as well as between the four different diameter definitions. For each model, the respective input diameter providing the best representation of the data is used in Figure 5, always annotating deviations from *d*_eq_.

Regardless of which model is employed, a certain scatter of predicted and measured velocities is evident from Figure 5. It is low with respect to the investigated fibers and reaches its maximum for the highly irregular PET fragments, which consistently score the lowest values of *R*_log_^2^, *R*^2^, and |AE| when comparing the different samples (see Table S3). Thus, the scatter can most likely be attributed to uncertainties in the determination of the size of individual MP fragments (also see the above section “Microplastic Fragments”) since particle size and density are the most sensitive input parameter of the velocity models.

There are clear differences in the performance of the tested models. In particular, models that were specifically proposed for MP particles (M1–M6, cf. Figure 5) cannot reliably predict the settling velocities of the small MPs investigated in this study. Among them, the model of Zhang and Choi^60^ (M4) provides the best representation, yet it still appears to systematically overestimate the lower settling velocities measured for smaller particles. This trend is even more pronounced for the models of Yu et al.^59^ (M3) and Waldschläger and Schüttrumpf^47^ (M1)—predictions of the latter partially exceed corresponding measurements by 2 to 3 orders of magnitude, which is also reflected in negative coefficients of determination *R*_log_^2^ and *R*^2^ as well as a very high |*AE*|. These observations could be due to the fact that the discussed models were exclusively based on empirical data on larger MPs and therefore potentially fail to produce consistent results outside of that scope. Further particle size bias might be introduced into an empirical model if the underlying fitting procedure emphasizes larger particles and higher settling velocities, e.g., by solely focusing on the *R*^2^ or the root-mean-square error (RMSE).

With respect to the tested MP-specific models, only Goral et al.^40^ (M2) restrict their model’s application to a certain Reynolds number range—yet, when applied to the entire data, it even scores a higher *R*_log_^2^ than the models of both Yu et al.^59^ and Waldschläger & Schüttrumpf^47^ (see Table S2). Remarkably, even the empirical equation proposed by Kaiser et al.^43^ (M5) only provides poor predictions, although it was specifically derived from data on MPs of similar size. This poor predictive capability is probably due to experimental artifacts since their study features only few particles above 100 μm and partially inconsistent results for particles below 40 μm in size.^43^

In contrast, the integrity of the measurements from this study is further supported by the high accordance between the measured velocities and corresponding predictions from more general models (M7–M10, cf. Figure 5). The model of Bagheri and Bonadonna^61^ (M7) was previously confirmed to produce good results for large MPs^66^ and now performed superior with respect to small MPs, too. It is thus generally recommended for modeling the settling of nonbuoyant MPs—more so, as it features a concise and flexible parametrization of particle shape. Yet, using an alternative diameter definition notably proved beneficial for MP fibers (cf. Figure 3 and Table S4). The model of Su et al.^64^ (M10) performed almost as well in this study, yet applying it to large MPs revealed high sensitivity toward the choice of its shape parameter ε (cf. Section S8). Komar’s^63^ equations for ellipsoids and cylinders (M9) might be used, when exclusively modeling the terminal settling velocity of small MP fragments or fibers, respectively, as they scored very good values for *R*_log_^2^ and |*AE*| but are restricted to laminar flow. With respect to the discussed models, variations of water temperature and salinity can be implemented by changing density and viscosity, accordingly.

### Perspective for Future Research

This laboratory study on the settling of small, pristine MPs reveals that existing MP-specific formulas for computing terminal settling velocities, which are mainly based on MPs that are >500 μm in size, fail at predicting the settling velocities of smaller MPs. Still, most of these formulas are not complemented by a corresponding validity range, e.g., by means of the Reynolds numbers. The use of models outside of their actual scope can lead to severe error propagation: For instance, Bello et al.^67^ employed the model of Waldschläger and Schüttrumpf^47^ (M1) in order to derive probability distributions of terminal settling velocities of MPs between 20 and 300 μm in size. Regarding the considered particle size range, this implementation leads to overestimations of up to several orders of magnitude, as was already discussed with respect to Figure 5.

Fortunately, research on MPs can draw inspiration and insights from multiple other fields and continues to do so.^66,68^ This potential is also exemplified by the superior terminal settling velocity model tested in this study, which was developed in the context of volcanic ash particles, but has been abstracted sufficiently to allow for a more general application.^61^

Nevertheless, additional empirical data are needed in order to address the many open research questions still surrounding the environmental fate of MPs. In natural waters, vertical transport of MPs is of course governed not only by the flow conditions and particle properties that determine their nominal settling velocities as investigated in this study but also by changes in these particle properties brought on by weathering, interactions with other particles and biota, and aggregation.^8^ Several studies, mostly focusing on large MPs, have already been conducted to address such phenomena.^13,32,35,69,70^ This study and the employed measuring setup^48^ provide an example for measuring settling velocities with high accuracy, which might be adopted in future experiments in order to characterize and quantify further influence factors on the settling of small MPs. Yet, the processes in question can be highly multidimensional, e.g., including concentration dependencies, variations of environmental conditions, and specific effects related to the size or shape of MPs. Therefore, it is even more necessary to constantly integrate new experimental results into the existing scientific discourse: for instance, by proposing corrections or expansions of validated frameworks for computing settling or rising velocities in order to include additional effects instead of providing new formulations that are exclusively based on small, specific data sets. Additionally, underlying data should be provided entirely and as transparent as possible in order to enable future reassessments and thus significantly increase its value.

## Data Availability

Single-particle raw data of all experiments, a manual for producing fibers of defined length via cryosectioning and corresponding measurements of fiber lengths from microscopy images, are provided in the Zenodo repository “Additional data for Settling velocities of small microplastic fragments and fibers” at 10.5281/zenodo.10049926 (DOI).

## Supporting Information Available

All scanning electron microscopy images, results and discussion of filtering procedures after particle tracking, computation of size and shape descriptors, details on the implementation of each tested terminal settling velocity model, computation of a fiber’s inclination angle, measured drag coefficients, and entire results from comparing all terminal settling velocity models (PDF)

## Abbreviations

ECD: equivalent circular diameter
FOV: field of view
Mt: metric tons
MP: microplastic
PA 6.6: polyamide 6.6
PET: poly(ethylene terephthalate)
PMMA: poly(methyl methacrylate)
PS: polystyrene
PVC: poly(vinyl chloride)
PUR: polyurethane
RMSE: root-mean-square error
ROI: region of interest
SEM: scanning electron microscopy
