# Correction to “Settling Velocities of Small Microplastic Fragments and Fibers”

**DOI:** 10.1021/acs.est.5c08557

**Published:** 2025-07-24

**Authors:** Stefan Dittmar, Aki S. Ruhl, Korinna Altmann, Martin Jekel

Unfortunately, the implementation/coding of one of the 10 settling velocity models tested in this paper (Yu et al., M3) was erroneous. In fact, the model provides more accurate results for predicting the settling velocities of small MPs than originally stated. Additionally, eq 4 contained a typographical error. We sincerely apologize for these errors and thank Prof. Matthew Hoffmann, who worked with the openly available raw data (doi.org/10.5281/zenodo.10049926), and Dr. Asmus A. Meyer-Plath for noting them.

The overall conclusions and recommendations of this paper are not affected, since the highlighted general settling velocity models still outperform MP-specific models. All changes are mentioned below.

The respective panel (M3: Yu) in Figure 5 has been corrected.

**Figure 5:**
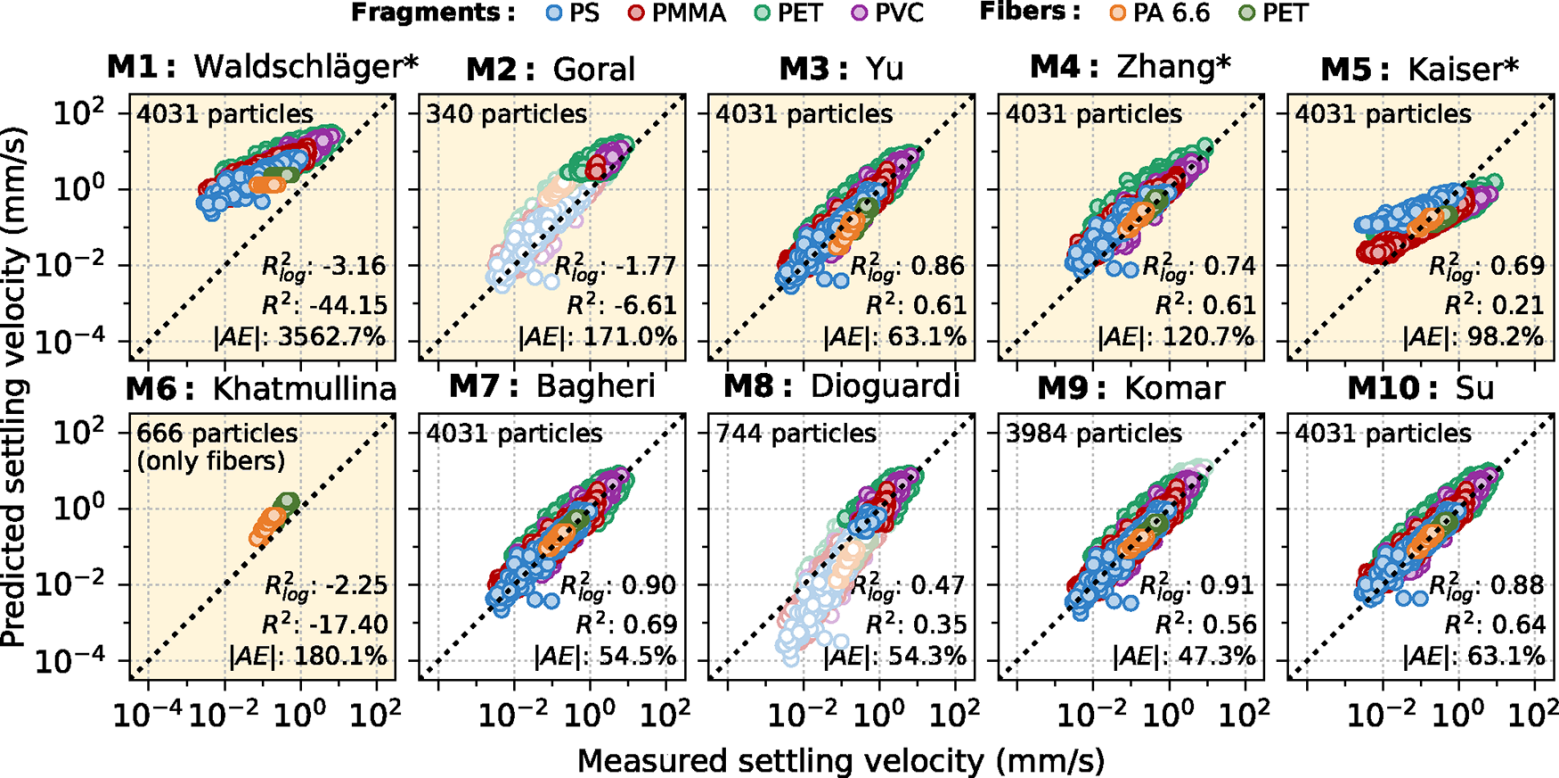
Measured settling velocities versus corresponding predictions by each of the tested terminal settling velocity models (note the log–log scale). Performance measures *R*_log_^2^, *R* ^2^, and |AE| are indicated, respectively. If a validity range is specified for a model according to the Reynolds number, outliers are not considered and depicted pale. Use of *d*_2_ instead of *d*_eq_ as input diameter is indicated by * (see section S7). Models derived specifically for MP are highlighted with a yellow background.

Consequently, the changes to the text (in **bold**) are listed in Table 1.

**Table 1:** Corrections to the Main Text (Changes Are Highlighted in Bold)

page	original	corrected
6359	“It is concluded that models, which were specifically deduced from empirical data on larger microplastics, fail to provide accurate predictions for small microplastics.”	“It is concluded that models, which were specifically deduced from empirical data on larger microplastics, **mostly** fail to provide accurate predictions for small microplastics.”
6362	4 $d_{\mathrm{eq.MP}\;\mathrm{fiber}}=\sqrt{1.5D^{2}L}$	4 $d_{\mathrm{eq.MP}\;\mathrm{fiber}}=\sqrt[{3}]{1.5D^{2}L}$
6365	“In particular, models that were specifically proposed for MP particles (M1–M6, cf. Figure 5) cannot reliably predict the settling velocities of the small MPs investigated in this study. Among them, the model of Zhang and Choi^60^ (M4) provides the best representation, yet it still appears to systematically overestimate the lower settling velocities measured for smaller particles. This trend is even more pronounced for the models of Yu et al.^59^ (M3) and Waldschläger and Schüttrumpf^47^ (M1)—predictions of the latter partially exceed corresponding measurements by 2–3 orders of magnitude, which is also reflected in negative coefficients of determination *R*_log_^2^ and *R* ^2^ as well as a very high |AE|.”	“In particular, models that were specifically proposed for MP particles (M1–M6, cf. Figure 5) cannot reliably predict the settling velocities of the small MPs investigated in this study. **Among them, the model of Yu et al.^59^ (M3) achieves the best representation and provides accurate predictions for MP fragments yet not for fibers. The model of Zhang and Choi^60^ (M4)** appears to systematically overestimate the lower settling velocities measured for smaller particles. This trend is even more pronounced for **the model of** Waldschläger and Schüttrumpf^47^ (M1)—**its predictions** partially exceed corresponding measurements by 2–3 orders of magnitude, which is also reflected in negative coefficients of determination *R*_log_^2^ and *R* ^2^ as well as a very high |AE|.”
6365	“With respect to the tested MP-specific models, only Goral et al.^40^ (M2) restrict their model’s application to a certain Reynolds number range—yet, when applied to the entire data, it even scores a higher *R*_log_^2^ than the models of both Yu et al.^59^ and Waldschläger and Schüttrumpf^47^ (see Table S2).”	“With respect to the tested MP-specific models, only Goral et al.^40^ (M2) restrict their model’s application to a certain Reynolds number range—yet, when applied to the entire data, it even scores a higher *R*_log_^2^ than the **model of** Waldschläger and Schüttrumpf^47^ (see Table S2).”
6366	“This laboratory study on the settling of small, pristine MPs reveals that existing MP-specific formulas for computing terminal settling velocities, which are mainly based on MPs that are >500 μm in size, fail at predicting the settling velocities of smaller MPs.”	“This laboratory study on the settling of small, pristine MPs reveals that existing MP-specific formulas for computing terminal settling velocities, which are mainly based on MPs that are >500 μm in size, **mostly** fail at predicting the settling velocities of smaller MPs.”

The Supporting Information file was also updated accordingly. Corrected plot panels for Yu et al. (model M3) in Figure S17. Corrected performance measures for Yu et al. (model M3) in Tables S2–S4. In section S6, which is no subject of the main text, an additional update now includes a specific calculation of drag coefficients for MP fibers (based on length, diameter, and inclination instead of just equivalent diameter as for fragments). The equations and Figures S15 and S16 were updated accordingly. The data set “2_drag_coefficients.zip” (in the Zenodo repository doi.org/10.5281/zenodo.10049926) was also updated.

## Supplementary Material



## Notes

All scanning electron microscopy images, results and discussion of filtering procedures after particle tracking, computation of size and shape descriptors, details on the implementation of each tested terminal settling velocity model, computation of a fiber’s inclination angle, measured drag coefficients, and entire results from comparing all terminal settling velocity models (PDF)

