# Supplementary material for: Settling Velocities of Small Microplastic Fragments and Fibers
Source: Environ Sci Technol. 2024 Mar 21;58(14):6359–69. doi: 10.1021/acs.est.3c09602 (PMC11008250; doi:10.1021/acs.est.3c09602)
Supplement: Supplementary file 1 — es3c09602_si_001.pdf [file es3c09602_si_001.pdf]

SUPPORTING INFORMATION

Settling Velocities of Small Microplastic Fragments and Fibers

Stefan Dittmar,*[,†,‡] Aki S. Ruhl,[†,§] Korinna Altmann,[||] Martin Jekel[†]

[†] Technische Universität Berlin, Chair of Water Quality Control,
Str. des 17. Juni 135, 10623 Berlin, Germany

[‡] GEOMAR Helmholtz Centre for Ocean Research Kiel,
Wischhofstraße 1–3, 24148 Kiel, Germany

[§] German Environment Agency (UBA), Section II 3.3,
Schichauweg 58, 12307 Berlin, Germany

[||] Bundesanstalt für Materialforschung und -prüfung (BAM),
Unter den Eichen 87, 12205, Berlin, Germany

* Corresponding author: stefan.dittmar@tu-berlin.de

Number of pages: 32
Number of figures: 21
Number of tables: 4

Content

S1 Scanning electron microscope images 3

S2 Filtering particle tracking results 6

S2.1 Particle size cutoffs 6

S2.2 Visual inspection 6

S2.3 Applying empirical model for particle-particle interactions 9

S3 Particle size and shape descriptors 14

S4 Implementation of terminal settling velocity models 16

S4.1 Waldschläger & Schüttrumpf (2019) 16

S4.2 Goral et al. (2023) 16

S4.3 Yu et al. (2022) 16

S4.4 Zhang & Choi (2022) 17

S4.5 Kaiser et al. (2019) 17

S4.6 Khatmullina & Isachenko (2017) 17

S4.7 Bagheri & Bonadonna (2016) 18

S4.8 Dioguardi et al. (2018) 18

S4.9 Komar (1980) 18

S4.10 Su et al. (2022) 19

S5 Inclination angle of fibers 20

S6 Measured drag coefficients 21

S7 Comparison of terminal settling velocity models 22

S8 Testing the model of Su et al.[18] for large microplastics 29

S9 References 31

S1 Scanning electron microscope images

Scanning electron microscope (SEM) images of all investigated particles were acquired on a Gemini DSM 982 (Zeiss, Germany) equipped with DISS6 (point electronic, Germany) and are partly shown in the original publication. The following tableaus comprise the images of microplastic (MP) fragments (Figure S1), PA 6.6 fibers (Figure S2) and PET fibers (Figure S3), respectively. Each sample is depicted at three different magnifications as indicated by scale bars.

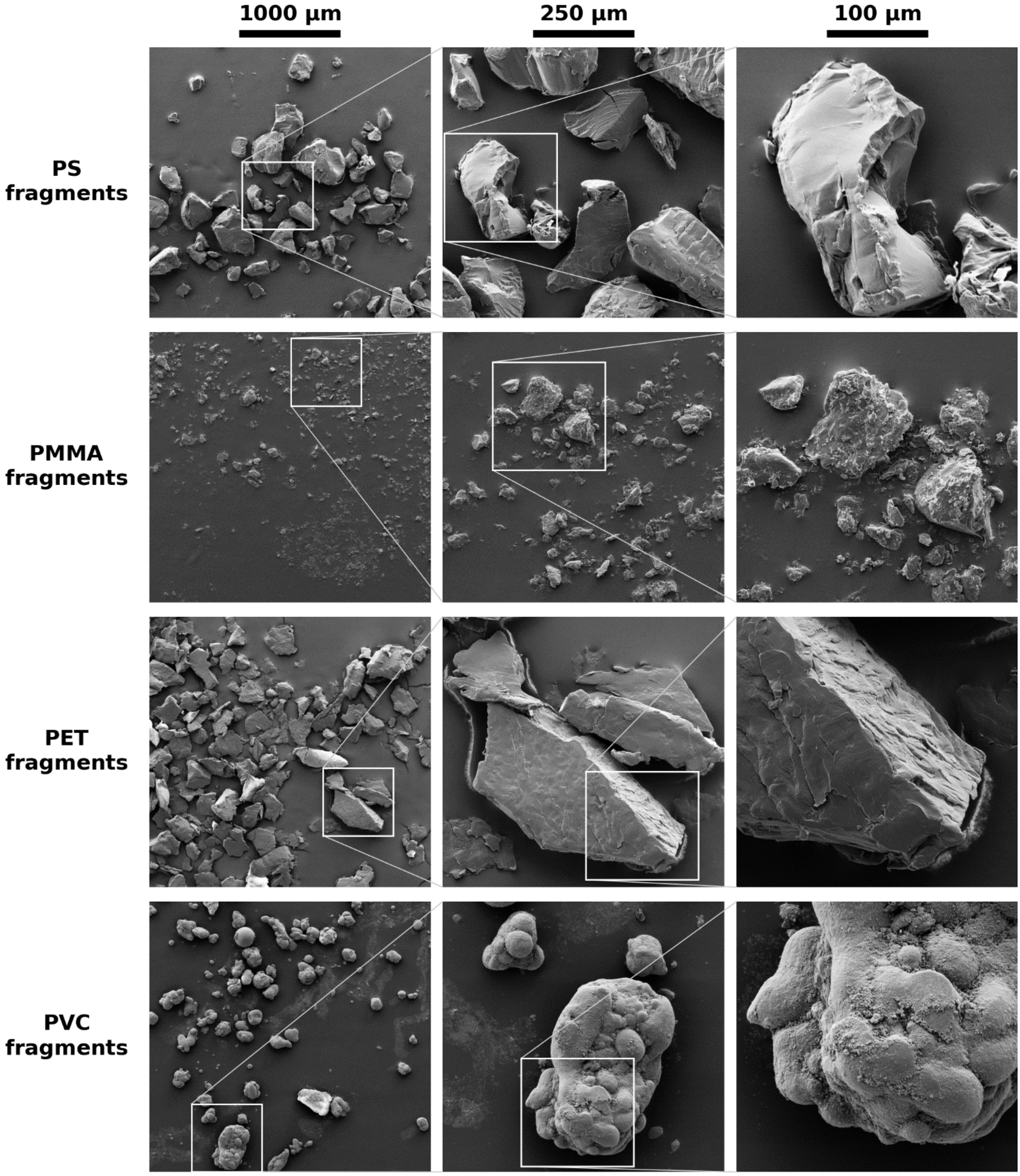

Figure S1. Exemplary SEM images of all investigated MP fragments. Note magnifications (columns).

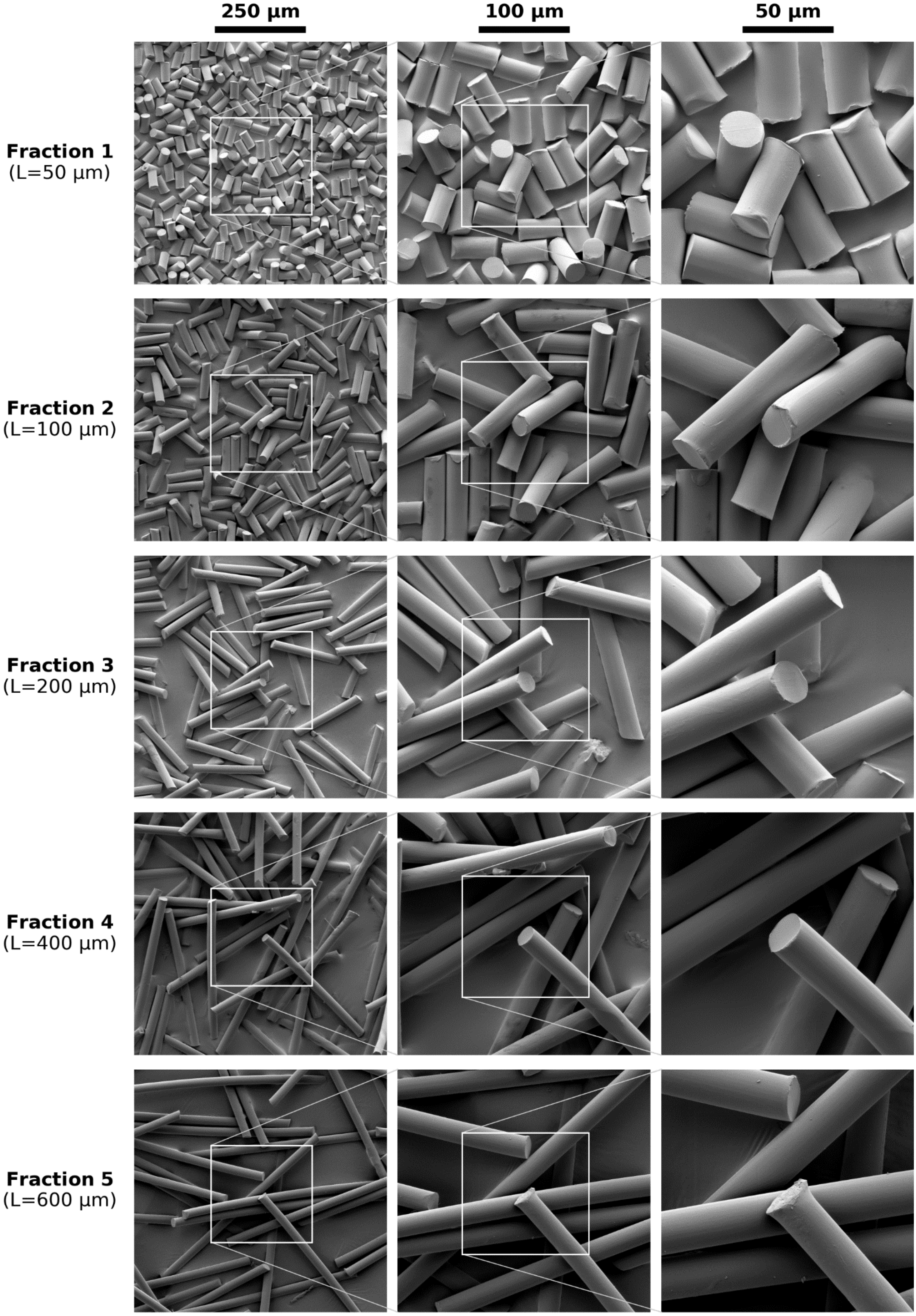

Figure S2. SEM images of length fractions of PA 6.6 fibers. Note magnifications (columns).

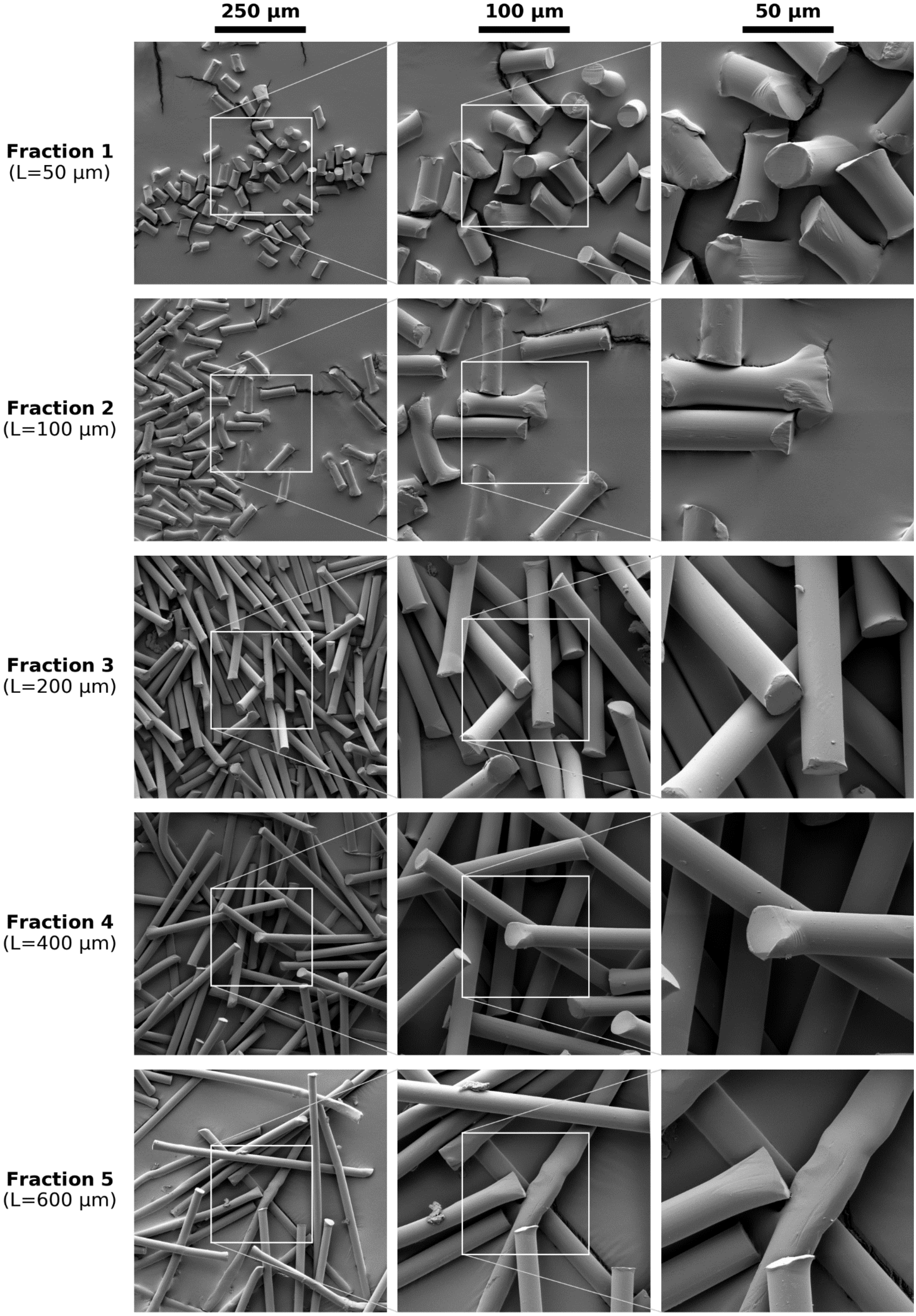

Figure S3. SEM images of length fractions of PET fibers. Note magnifications (columns).

S2 Filtering particle tracking results

S2.1 Particle size cutoffs

Settling experiments with MP fragments were conducted in several sets with gradually reduced runtime in order to measure an increased number of larger MP particles. The runtime of the first set of experiments was fixed based on a longer, preliminary run with the respective samples. It was chosen to be long enough to capture even the sample's particles with the lowest settling velocity. Usually this corresponds to the smallest size fraction of the sample, while always acknowledging the lower size limit for particle detection of 10 µm, which was previously described for this setup by Dittmar et al.[1].

Reducing the duration of an experimental run thus leads to a carryover of slow-settling, mostly smaller particles that were dosed during previous runs. As sufficient numbers of these particles were already measured during sets of experiments with increased runtimes, they were generally excluded from further evaluation by introducing respective size cutoffs. More so, as their settling velocities are probably affected by interactions with larger, recently dosed particles. This is also apparent from Figure S8 and Figure S9, which depict measured particles below the size cutoffs as well – a significant proportion of which does not comply with criteria set for the maximum modeled velocity deviation (see section S2.3). Some measured velocities even notably exceed Stokes' law and the Kaskas equation[2], which are plotted for the considered particle size range and density, respectively. Both equations describe the theoretical settling velocities of a sphere, with Stokes' law being only valid within the laminar flow regime.

The size cutoffs (cf. Table 1 of the main publication) refer to the equivalent circular diameter (ECD) of a particle and were based on the maximum size of all particles from sets of longer experimental runs, which were detected after the respectively considered runtime elapsed.

S2.2 Visual inspection

All particle tracking results were assessed by a human observer to identify and exclude potential agglomerates, contaminations or incomplete detections. Examples for the described categories are depicted in Figure S4. They were taken from data acquired during experiments with fibers. In each case, the region of interest (ROI) of the raw image as well as the detected contour is shown.

Some of the investigated polymer materials are translucent. Opposing particle surfaces can thus appear transparent on raw images if they are even and aligned in parallel to the image plane. Such transparencies occasionally prevent the image processing algorithm from capturing a particle contour in its entirety. Yet, these incomplete detections occurred only rarely, e.g. in some cases in which fibers are aligned orthogonal to the image plane as it is shown in Figure S4. Regarding MP fragments, less than 1% of all particles were not detected completely – often even at just one of several frames. Only for PET fragments the number of incomplete detections is slightly increased (1.5%). It is evident from SEM images (cf. Figure S1) that the investigated PET fragments often exhibit a rather flat shape with even surfaces, which probably promotes such transparencies.

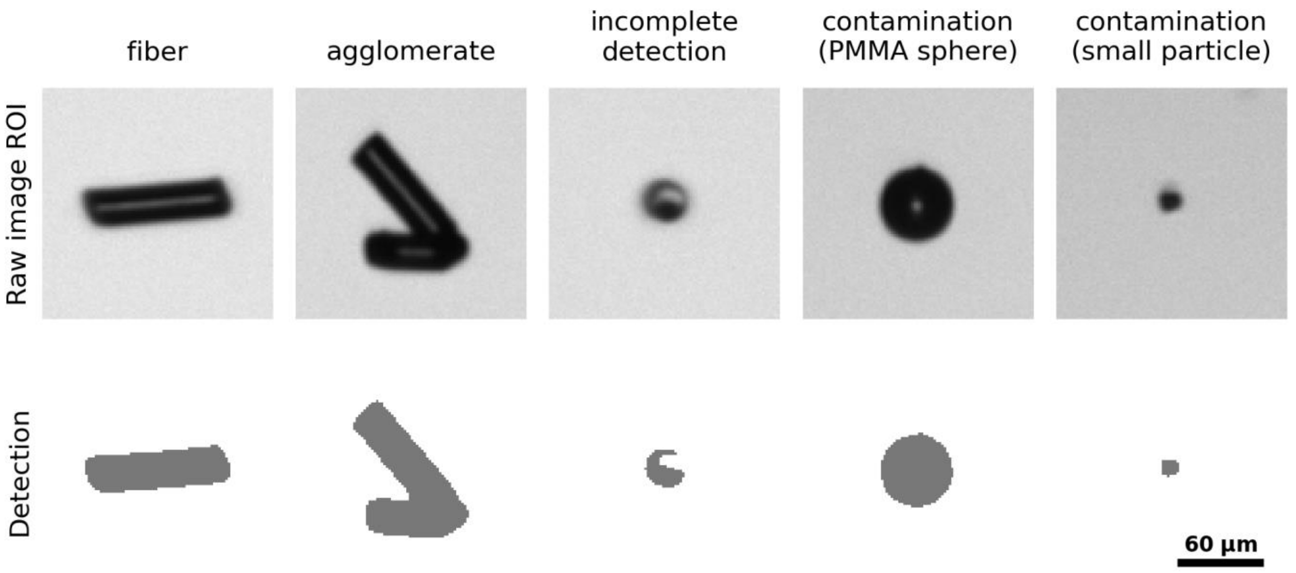

Figure S4. Exemplary visual classifications of particles from settling experiments with fibers (columns). The relevant section of the raw image (upper row) and the corresponding detected contour (lower row) are depicted, respectively. Note scale bar as well.

In compliance with partly imposed size cutoffs (cf. Table 2 of the main publication), 3603 particles have been successfully tracked during experiments with MP fragments. 31 of these particles (0.9%) were not further considered after visual inspection due to incomplete detection (28 particles) or due to being agglomerates (3 particles). No particles were identified as contaminations. Although the irregular shapes of fragments would only allow to spot prominent contaminations, e.g. large fibrous particles, a negligible contamination rate is very likely and in line with previous results: Validation experiments with spherical and thus distinctive particles already revealed a mean contamination rate of only 0.5% using the identical measuring setup[1].

With respect to results from experiments with MP fibers, the number of particles excluded after visual inspection is notably higher. In total, 947 particle trajectories were obtained, 187 of which (19.7%) had to be rejected – either as contaminations (114 particles, 12.0%), agglomerates (58 particles, 6.1%) or due to incomplete contour detection (15 particles, 1.6%). Classification results of all investigated length fractions are depicted in detail in Figure S5 for PA 6.6 fibers and in Figure S6 for PET fibers, respectively.

A classification as agglomerate must not only be caused by insufficient stabilization of particles, but could instead originate from fiber cutting artifacts that connected two filaments. It might as well be the observed joint settling of fibers without actual adhering forces, which probably occurs more regularly with increased fiber length and would thus be consistent with the trend visible in Figure S5 and Figure S6. Only 1.6% of all fibers were incompletely detected in at least one frame and were thus excluded from further evaluation, too. It could be argued, that incomplete detections have a minor impact in the case of fibers, because uniform length is assumed for every investigated fraction anyway. Still, the exclusion was done analogously to the handling of results from experiments with MP fragments.

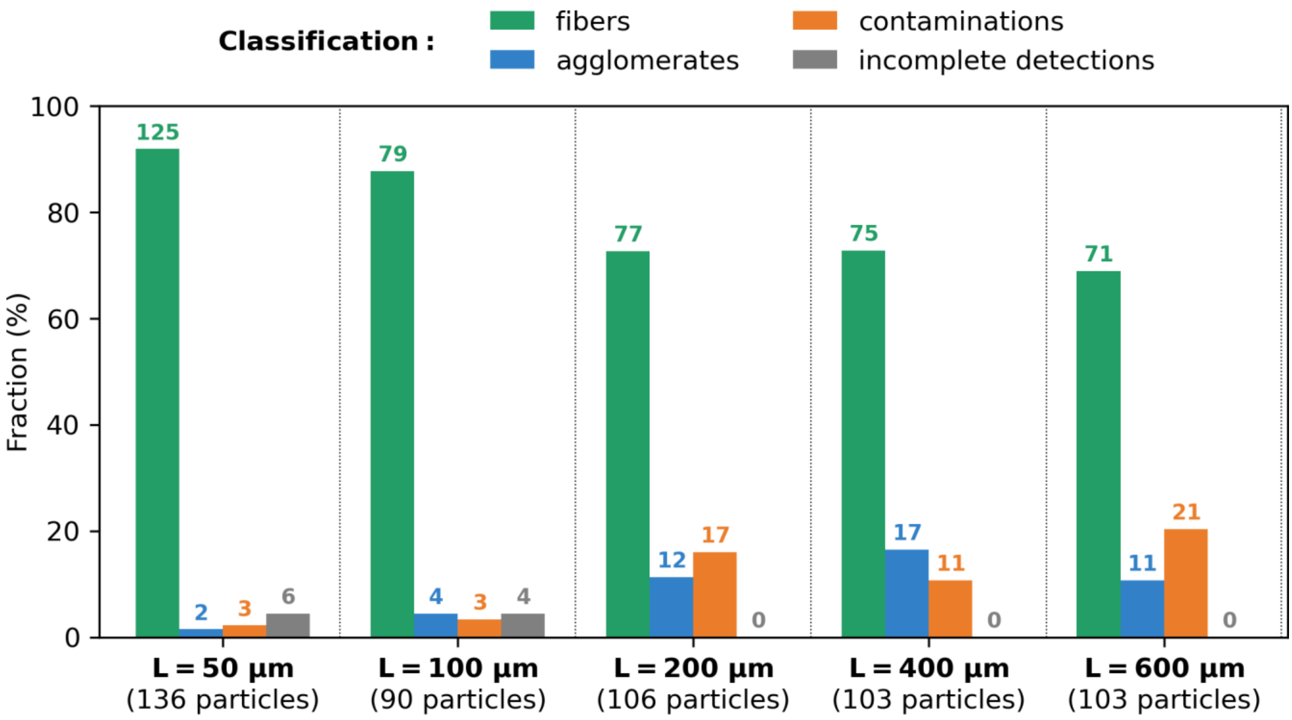

Figure S5. Results of visual inspection of particles detected in settling experiments with PA 6.6 fibers. Relative fractions of different classification options are plotted as bars for each investigated length fraction. Absolute numbers of classified particles are indicated above the bars, respectively.

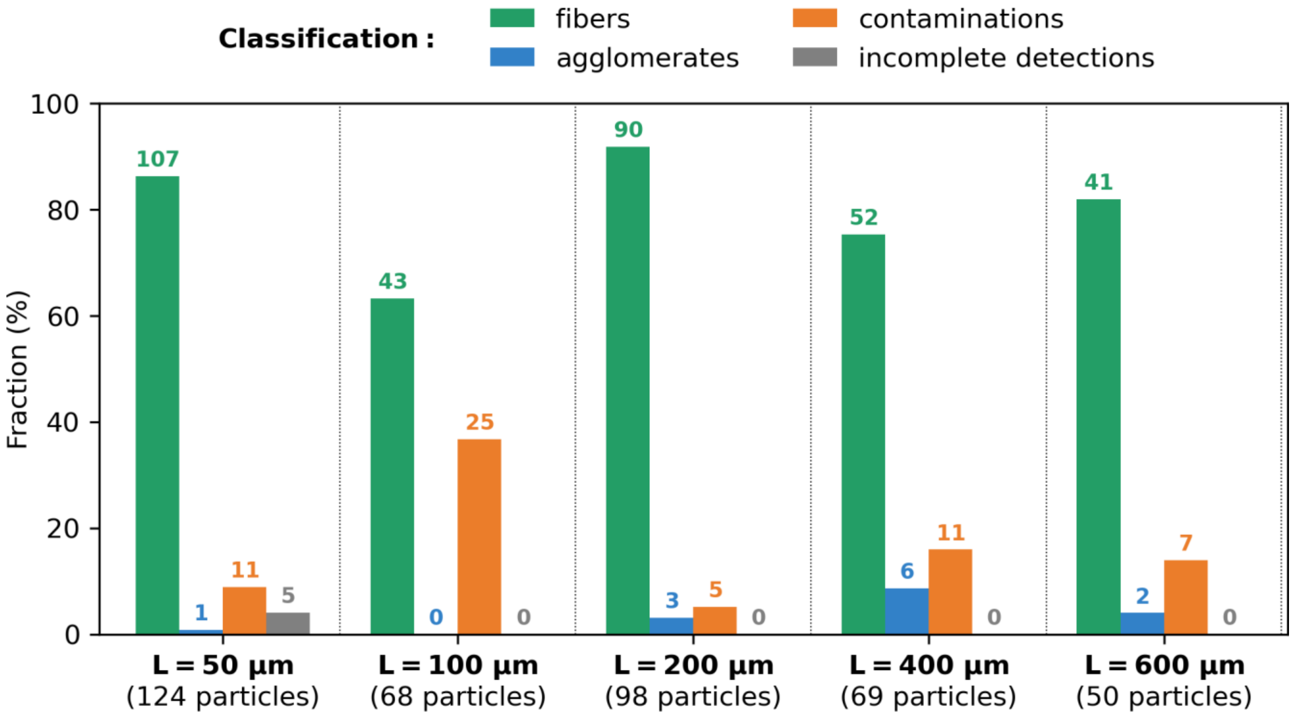

Figure S6. Results of visual inspection of particles detected in settling experiments with PET fibers. Relative fractions of different classification options are plotted as bars for each investigated length fraction. Absolute numbers of classified particles are indicated above the bars, respectively.

Compared to validation experiments with spherical particles (0.5%)[1] – which can be visually well distinguished from other particles, too – a substantially larger share of particles was classified as contaminations during experiments with fibers (12.0%). Yet, there are various reasons for this increase: On the one hand, the applied particle doses were very low (18.5 µg) in order to account for monodispersity and minimize interactions between the settling fibers[1]. Thus, the proportion of particle contaminations versus targeted fibers potentially increases. Yet more importantly, the protocol for embedding and cutting fibers is laborious (cf. instructions available from the Zenodo repository[3] or original protocol by Cole[4]) and consequently involves several potential sources of particle contaminations. Despite minimizing airborne contamination by covering samples as much as possible during preparation and fiber embedding, contamination cannot be fully prevented – especially as the protocol requires intensive handling of samples and many cutting operations. The obtained cryosections have to be suspended to fully dissolve the embedding matrix and thus not only recover target particles but potential contaminations, too.

The experiment with 100 µm long PET fibers was additionally contaminated with several PMMA spheres (cf. example given in Figure S4 and respective fraction in Figure S6) from an immediately preceding settling experiment. Although the settling column and dosing funnel was always cleaned intensively between experiments as detailed in a previous publication[1], the previously applied doses of PMMA spheres up to 500 mg were extremely high and apparently caused the carryover of some particles. Yet, the situation was manageable, since the fibers are distinct and thus enable impeccable identification of contaminations. The problem was fixed with the subsequent cleansing and was exceptional in the sense that the concerned doses of PMMA spheres were between 2 and 5 orders of magnitude above the doses used in every other conducted experiment.

S2.3 Applying empirical model for particle-particle interactions

The conducted experiments aim at measuring the settling velocities of single particles. Yet, in order to handle particles of the targeted size, they have to be dosed in suspension – thus allowing interactions between settling particles, which potentially affect measured velocities. This was already demonstrated for spherical particles of varying density (1.05–2.46 g/cm^3) using the identical experimental setup. An empirical model was based on this data, which describes the increase of a particle's settling velocity due to interactions with other particles.[1] This model was now applied to all data from experiments with MP fragments and fibers. It does not only consider particles, which are in-focus, but all particles that are initially tracked via image processing. Details are given in the original publication[1] and its Supporting Information. For each target particle, the deviation from its nominal single particle settling velocity was computed as model output. Although this predicted deviation is not accurate enough to directly correct measurements (e.g. because the model was only calibrated with spheres), it indicates the quality of the respective measurement – especially if strict criteria are applied. Therefore, any measurement of a particle, which exceeds a predicted absolute velocity deviation of 0.01 mm/s as well as a relative deviation of 5% with respect to the actually measured velocity, was not considered. In total, this excluded 94 MP fibers (12.4%) and 207 MP fragments (5.8%) from further evaluation. Details are depicted in Figures S7–S12. Overall, the proportion of excluded measurements is mostly low, indicating a good choice of particle doses. For Set 3 of PMMA fragments, a reduced dose might have been beneficial.

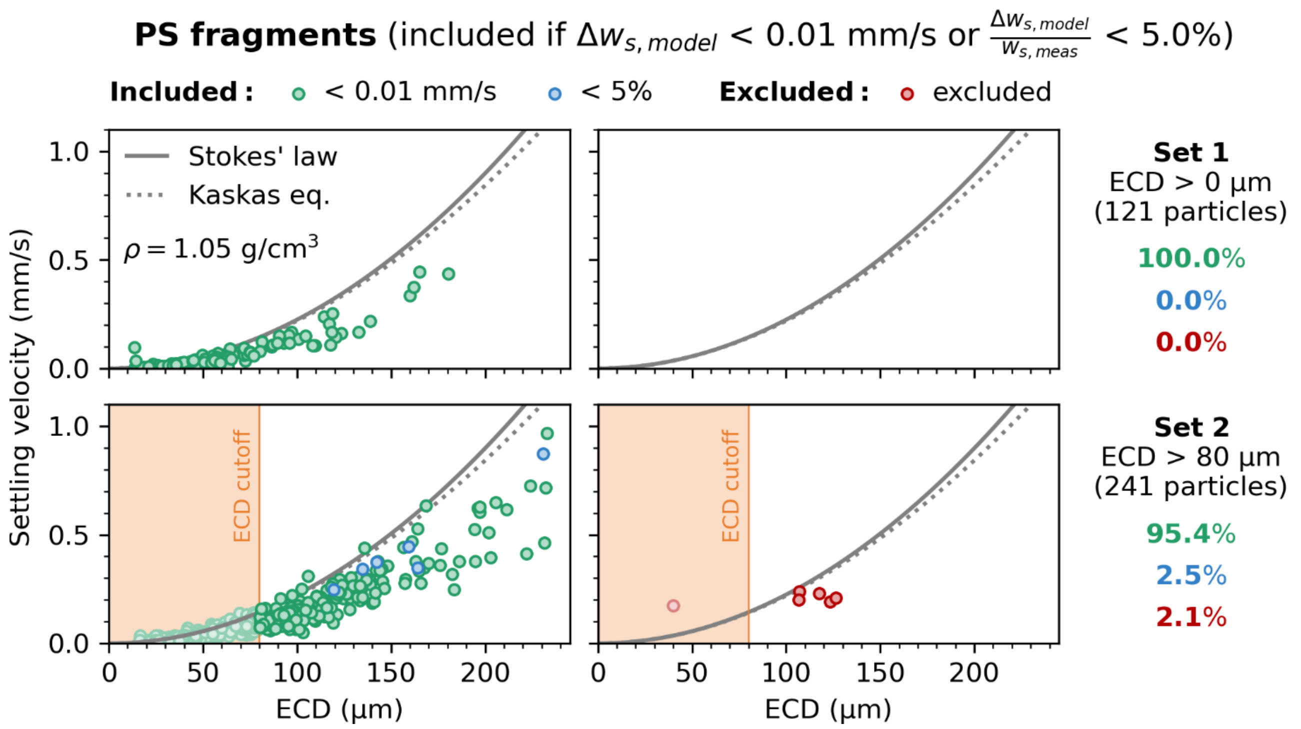

Figure S7. PS fragments with criteria for modeled deviation from single particle settling velocity.

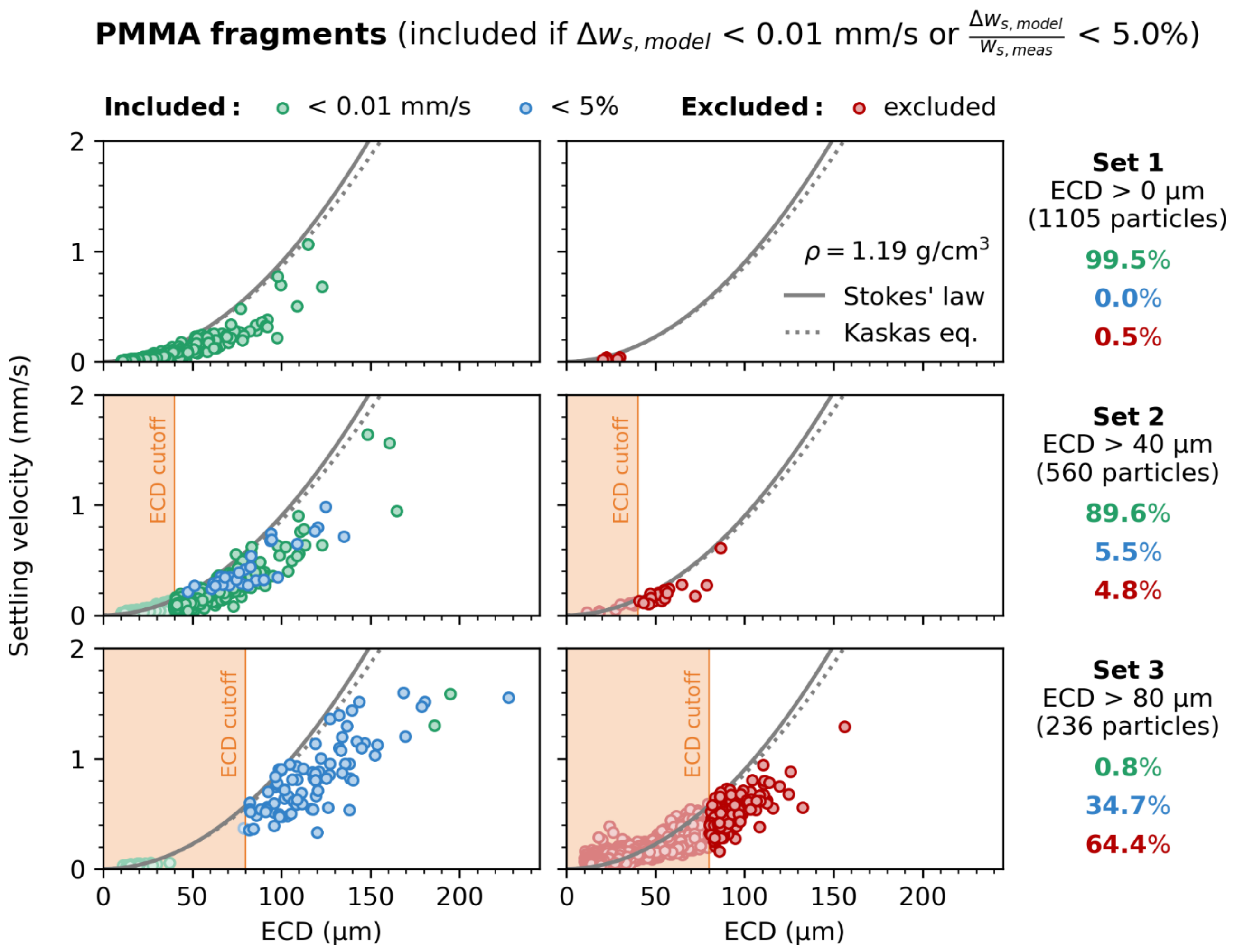

Figure S8. PMMA fragments with criteria for modeled deviation from single particle settling velocity.

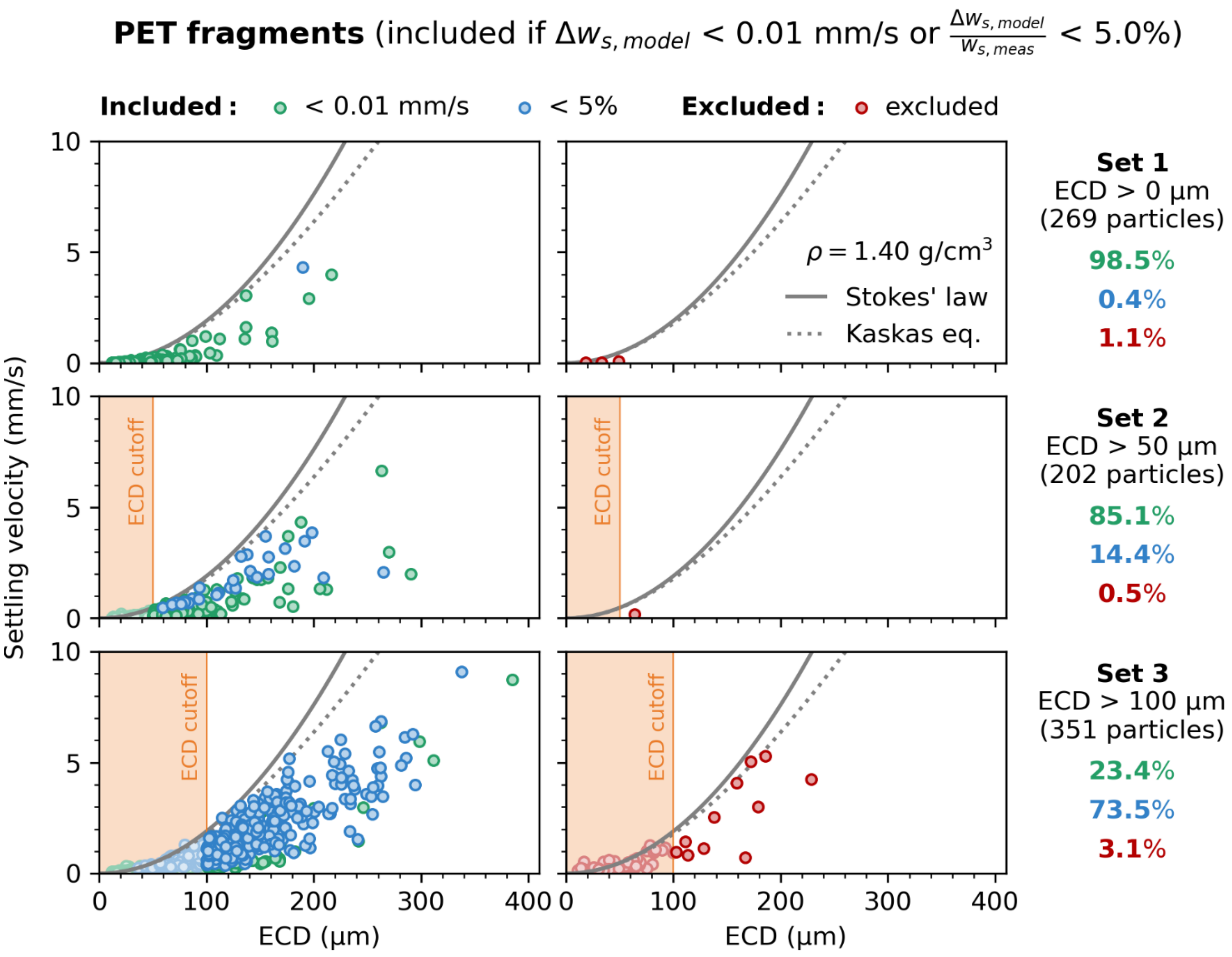

Figure S9. PET fragments with criteria for modeled deviation from single particle settling velocity.

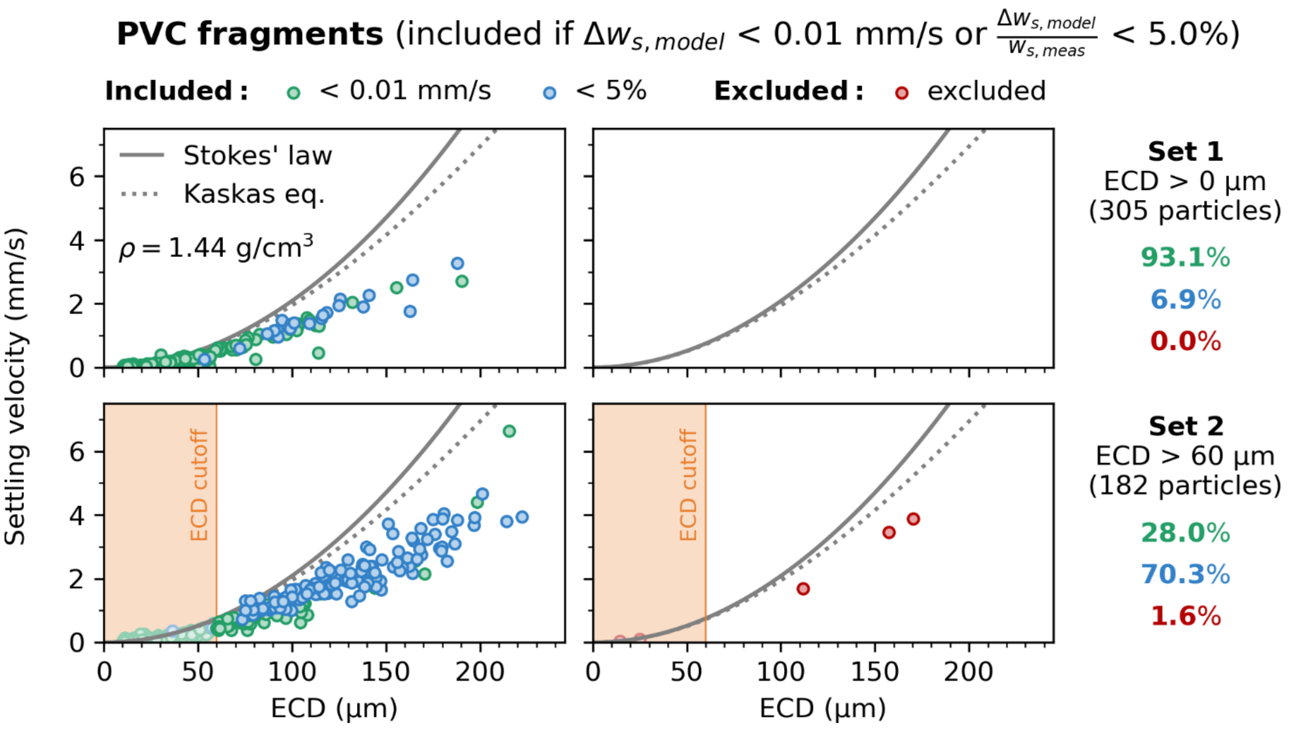

Figure S10. PVC fragments with criteria for modeled deviation from single particle settling velocity.

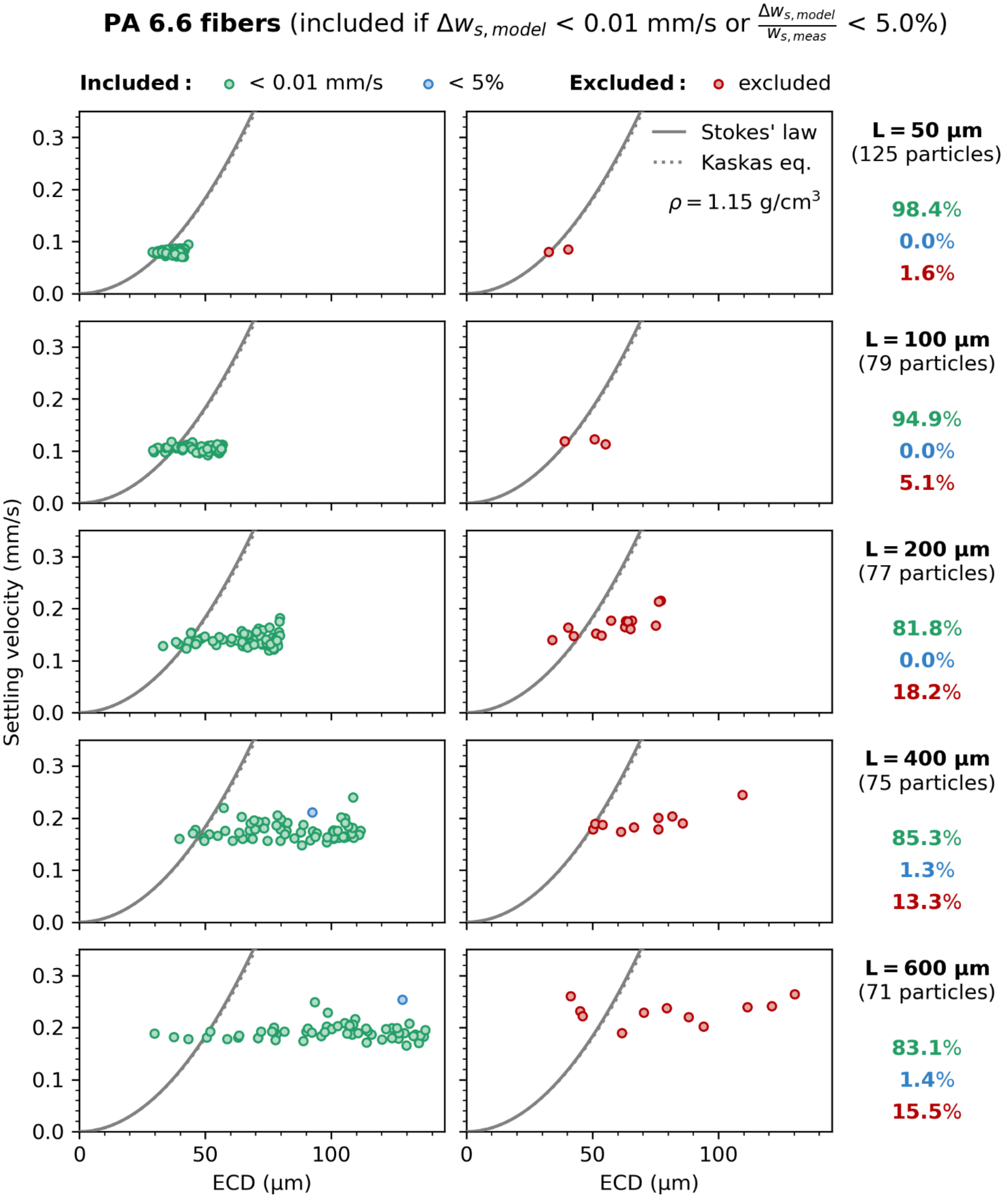

Figure S11. Detected PA 6.6 fibers with applied criteria for modeled deviation from single particle settling velocity. All investigated length fractions (50, 100, 200, 400 or 600 µm, respectively) shown as indicated on the right hand-side.

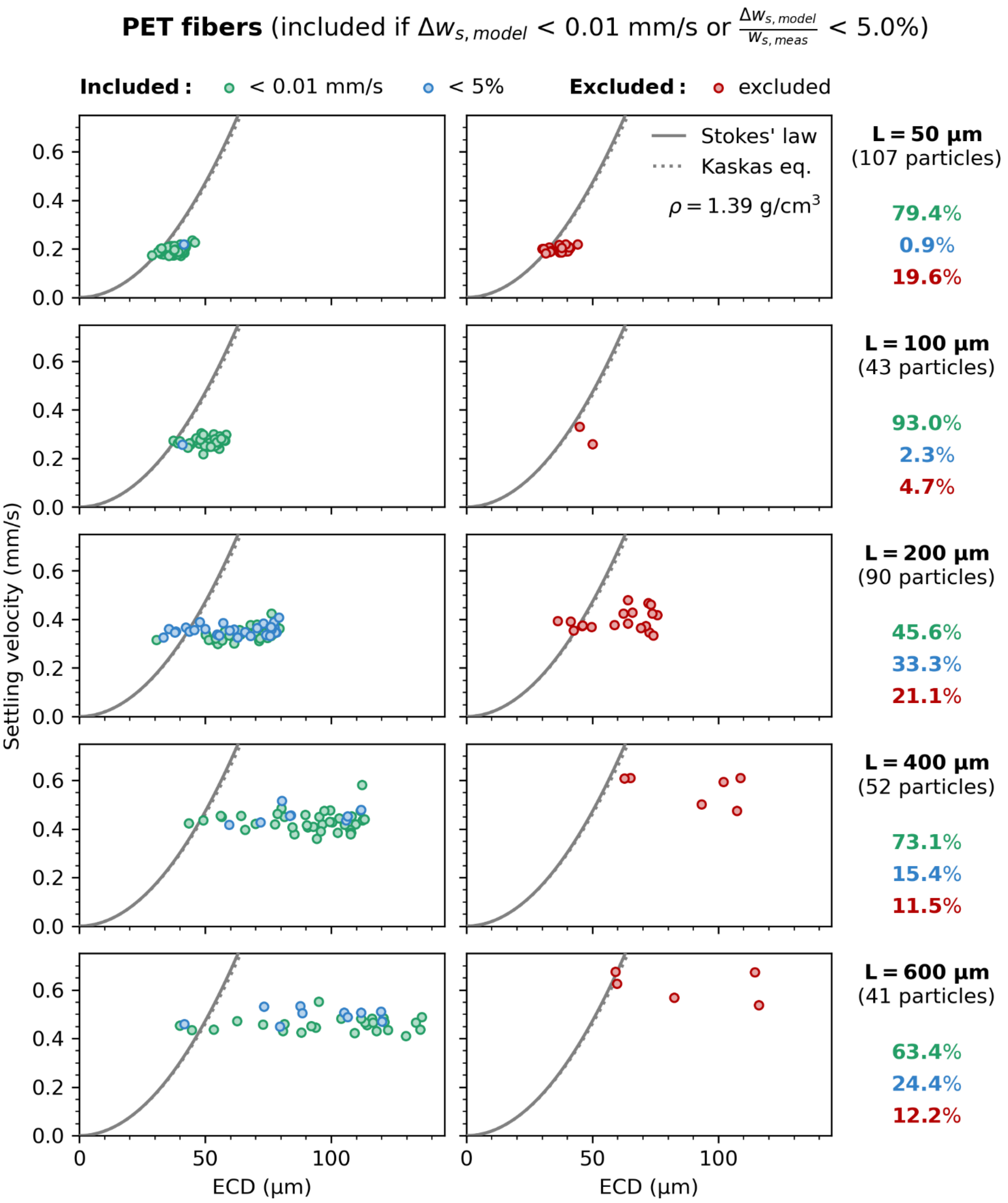

Figure S12. Detected PET fibers with applied criteria for modeled deviation from single particle settling velocity. All investigated length fractions (50, 100, 200, 400 or 600 µm, respectively) shown as indicated on the right hand-side.

S3 Particle size and shape descriptors

Implementing theoretical formulas for predicting a particle's terminal settling requires to parameterize its size and shape. The tested models (see section S4) presuppose a number of different descriptors for size and shape. The small size of the MP fragments investigated in this study prevents to conduct separate settling experiments with individual particles including a preceding characterization of their size and shape as it has been done for large MP.[5–7] Instead, the MP fragments are dosed in suspension. For each tracked MP fragment, size and shape descriptors have thus to be estimated solely based on the respective particle contours obtained from the raw images.

For a given contour Ci, its area A_i, the circularity $f_{circ,i}$ as well as length l and width w of the fitted rotated bounding rectangle with minimum area are extracted. $f_{circ,i}$ is computed following an area-based approach previously described by Dittmar et al.[1] (see section S6 of the respective Supporting Information). This circumvents discretization artifacts that occur when determining the perimeter of a pixel contour.[8] A correction of $f_{circ,i}$ to approximate a common perimeter-based circularity measure ($f^*_{circ,i} = 4\pi A_i/{P_i}^2$ with contour perimeter P_i) is adopted from Dittmar et al.[1] as well:

$$f^*_{circ,i} \cong \begin{cases} \frac{{f_{circ,i}}^2}{c}, & 0 < f_{circ,i} \leq c \\ 1 - \frac{(1-f_{circ,i})^2}{1-c}, & c < f_{circ,i} \leq 1 \end{cases} \quad \text{with} \quad c = 0.935 \tag{S1}$$

A_i, $f^*_{circ,i}$, l_i and w_i of all contours serve as a starting point for estimating the necessary size and shape descriptors. Initially, the average circularity $\overline{f^*_{circ}}$ is calculated from all $f^*_{circ,i}$. Moreover, a mean diameter $\overline{d_{2D}}$ is computed from the A_i of three characteristic contours selected from all contours of the tracked particle – considering the contours with the smallest (A_{min}), the biggest (A_{max}) and the intermediate (A_{int}) contour area. A_{int} is determined as the contour area, which is closest to the average of A_{min} and A_{max}. $\overline{d_{2D}}$ is then given as the average of the respective equivalent circular diameters deduced from the contour areas:

$$\overline{d_{2D}} = \frac{\sqrt{4A_{min}/\pi} + \sqrt{4A_{int}/\pi} + \sqrt{4A_{max}/\pi}}{3} \tag{S2}$$

Consequently, $d_{eq,MP\,fragment}$ is computed following Bagheri et al.[9] (as already described in the main publication, cf. Equation 2):

$$d_{eq,MP\,fragment} = \frac{\overline{d_{2D}}}{1.022 \cdot \psi^{-0.29}} \cong \frac{\overline{d_{2D}}}{1.022 \cdot {\overline{f^*_{circ}}}^{-0.29}} \tag{S3}$$

Furthermore, the longest (d_l), intermediate (d_i) and shortest (d_s) axis lengths of a MP fragment are estimated as the minimum, mean and maximum of all l_i and w_i, respectively. The equivalent diameter of a sphere with identical surface area $d_{eq,A}$, that is required for computing the sphericity ϕ of MP fragments (cf. Table S1), is approximated from d_l, d_i and d_s. For this, the approach proposed by Dellino et al.[10] is employed, which was used for the model of Dioguardia et al.[11] as well:

$$d_{eq,A} = 2\left(\frac{(d_l/2)^z(d_i/2)^z + (d_l/2)^z(d_s/2)^z + (d_i/2)^z(d_s/2)^z}{3}\right)^{1/(2z)} \text{ with } z = 1.6075 \tag{S4}$$

With respect to MP fibers, discrete length fractions were investigated in separate experiments. Consequently, diameter D and mean lengths L were determined microscopically (cf. section ‘Particle samples’ of the main publication) and could be uniformly assumed for the measured fibers of each fraction. This approach is superior to pure image analysis: Due to the high aspect ratio of fibrous particles, successfully extracting their length L from 2D raw images is strongly dependent on their orientation to the image plane.

Assuming cylindrical shape with diameter D and mean length L, all size and shape parameters for MP fibers can now be calculated according to their respective original definitions (with length of intermediate axis $d_i = D$). For instance, the equivalent diameter d_{eq} is given as:

$$d_{eq,MP\,fiber} = \sqrt{1.5D^2L} \quad \text{(S5)}$$

Table S1 comprises the computation of all remaining descriptors for MP fragments and MP fibers, respectively. All models requiring a certain descriptor are indicated with reference to the respective subsection of section S4 that details their implementation.

Table S1. Computation of size and shape descriptors for MP fragments and MP fibers. Not mentioned before were: Particle surface (A_p), surface of a sphere of identical volume (A_{sph}), perimeter of maximum projection (P_{mp}) and perimeter of a circle of equivalent area (P_c).

**Descriptor**	**Computation for MP fragments**	**Computation for MP fibers**	**Models requiring descriptor (cf. section)**
Aschenbrenner shape factor, ASF	$ASF = \frac{d_l d_s}{{d_i}^2}$	$ASF = \frac{DL}{D^2} = \frac{L}{D}$	Zhang & Choi (S4.4)
Corey shape factor, CSF	$CSF = \frac{d_s}{\sqrt{d_i d_l}}$	$CSF = \frac{D}{\sqrt{DL}}$	Waldschläger & Schüttrumpf (S4.1), Yu et al. (S4.3)
Janke shape factor, E	$E = \frac{d_s}{\sqrt{\frac{{d_s}^2 + {d_i}^2 + {d_l}^2}{3}}}$	not required	Komar (S4.9)
Elongation, e	$e = \frac{d_i}{d_l}$	$e = \frac{D}{L}$	Bagheri & Bonadonna (S4.7), Su et al. (S4.10)
Flatness, f	$f = \frac{d_s}{d_i}$	$f = \frac{D}{D} = 1$	Bagheri & Bonadonna (S4.7), Su et al. (S4.10)
Sphericity, ϕ	$\phi = \frac{A_{sph}}{A_p} = \left(\frac{d_{eq}}{d_{eq,A}}\right)^2$	$\phi = \frac{A_{sph}}{A_p} = \frac{(1.5D^2L)^{2/3}}{DL + 0.5D^2}$	Yu et al. (S4.3), Dioguardi et al. (S4.8)
Circularity, X	$\mathrm{X} = \frac{P_{mp}}{P_c} \cong \frac{1}{\sqrt{f^*_{circ}}}$	$\mathrm{X} = \frac{P_{mp}}{P_c} = \frac{2(D+L)}{\sqrt{4\pi DL}}$	Dioguardi et al. (S4.8)
Shape factor, Ψ	$\Psi = \frac{\phi}{\mathrm{X}} \cong \frac{{d_{eq}}^2}{{d_{eq,A}}^2 \sqrt{f^*_{circ}}}$	$\Psi = \frac{\phi}{\mathrm{X}} = \frac{(1.5D^2L)^{2/3}\sqrt{4\pi DL}}{3D^2L + D^3 + 2DL^2}$	Dioguardi et al. (S4.8)
Aspect ratio, AR	$AR = \frac{d_l}{d_s}$	$AR = \frac{L}{D}$	not required by a model, but referred to in the main publication

S4 Implementation of terminal settling velocity models

S4.1 Waldschläger & Schüttrumpf (2019)

Waldschläger & Schüttrumpf[5] give separate expressions for the drag coefficients of MP fragments and MP fibers, respectively. Both require the Corey shape factor CSF (see Table S1):

$$C_{d,MP\,fragment} = 3\left(CSF \cdot \sqrt[3]{Re}\right)^{-1} \tag{S6}$$

$$C_{d,MP\,fiber} = 4.7/\sqrt{Re} + \sqrt{CSF} \tag{S7}$$

Opposed to the definitions for d_{eq} from the main publication, Waldschläger & Schüttrumpf[5] propose to use $d_{eq} = \sqrt[3]{d_s d_i d_l}$ for MP fragments and, most notably, the diameter D as $d_{eq} = D$ for MP fibers. Inserting d_{eq} and C_d into Equation 1 of the main publication enables the implicit calculation of w_s.

S4.2 Goral et al. (2023)

Goral et al.[7] present separate expressions for the drag coefficient of MP fragments and fibers:

$$C_{d,MP\,fragment} = \left(1 + \frac{3.2}{\sqrt{Re}} + \frac{32}{Re}\right) \cdot \min(0.44 \cdot \psi^{-2}, 1) \tag{S8}$$

$$C_{d,MP\,fiber} = \max(19 \cdot Re^{-0.6}, 0.86) \tag{S9}$$

The authors specify the Reynolds number Re to be > 0.4 or ≥ 1 to ensure the validity of Equation S8 or S9, respectively. The sphericity measure ψ, which is required in Equation S8, is approximated here as:

$$\psi = \left(\frac{{d_s}^2}{d_l d_i}\right)^{1/3} \tag{S10}$$

For MP fragments, Re is computed from the diameter $d = \sqrt{d_i d_l}$ instead of d_{eq}. Inserting the respective C_d into Equation 1 of the main publication enables the implicit calculation of w_s.

S4.3 Yu et al. (2022)

Yu et al.[12] propose a terminal settling velocity model based on the correction of the drag coefficient for a sphere of equivalent volume. First, a dimensionless diameter d_* is computed from d_{eq}:

$$d_* = d_{eq} \cdot \left(\frac{(\rho_p - \rho_f) \cdot g}{\rho_f \cdot \nu^2}\right)^{1/3} \tag{S11}$$

The drag coefficient of a sphere of equivalent volume ($C_{d,s}$) is calculated by:

$$C_{d,s} = \frac{432}{{d_*}^3}\left(1 + 0.022{d_*}^3\right)^{0.54} + 0.47 \cdot \left(1 - \exp\left(-0.15{d_*}^{0.45}\right)\right) \tag{S12}$$

To account for particle shape, C_d is derived from $C_{d,s}$ using Corey shape factor CSF and sphericity ϕ (cf. Table S1):

$$C_d = \frac{C_{d,s}}{\left(d_*^{\beta_1} \phi^{d_*^{\beta_2}} CSF^{d_*^{\beta_3}}\right)^{\beta_4}} \tag{S13}$$

With the calibrated parameters $\beta_1 = -0.25$, $\beta_2 = 0.03$, $\beta_3 = 0.33$ and $\beta_4 = 0.25$. The terminal settling velocity w_s is now obtained as follows:

$$w_s = \left(\nu g \frac{\rho_p - \rho_f}{\rho_f}\right)^{1/3} \cdot \sqrt{\frac{4d_*}{3C_d}} \tag{S14}$$

S4.4 Zhang & Choi (2022)

Zhang & Choi[13] present a general drag coefficient for MP fragments, fibers and films based on the Aschenbrenner shape factor ASF (cf. Table S1):

$$C_d = 58.58 \cdot ASF^{0.1936} / Re^{0.8273} \tag{S15}$$

The authors decided to compute the equivalent diameter d_{eq} from the maximum projection area as:

$$d_{eq} = \sqrt{4 d_l d_i / \pi} \tag{S16}$$

Inserting C_d into Equation 1 of the main publication allows the implicit calculation of w_s.

S4.5 Kaiser et al. (2019)

Kaiser et al.[14] proposed an empirical equation for predicting settling velocities of small MP fragments:

$$w_s = 11.68 + 0.1991 \cdot d_{eq} + 0.0004 \cdot {d_{eq}}^2 - 0.0993 \cdot \Delta\rho + 0.0002 \cdot \Delta\rho^2 \tag{S17}$$

with $\Delta\rho = \rho_p - \rho_f$. In order to implement this formula properly, the units have to be used according to the authors. They express d_{eq} in [µm] and $\Delta\rho$ in [kg/m³] in order to compute w_s in [m/d]. Furthermore, they propose to compute d_{eq} as:

$$d_{eq} = \sqrt[3]{{d_s}^2 d_l} \tag{S18}$$

S4.6 Khatmullina & Isachenko (2017)

Khatmullia & Isachenko[15] derived the following empirical equation for computing the settling velocities of MP fibers from length D and diameter L:

$$w_s = \frac{\pi \cdot g \cdot (\rho_p - \rho_f)}{2 \cdot \nu \cdot \rho_f g} \cdot \frac{DL}{L \cdot 5.5283 \cdot 10^4 + 12.691} \tag{S19}$$

One coefficient was originally given as 55.283 mm^{-1}, but was converted to SI units here for conformity reasons.

S4.7 Bagheri & Bonadonna (2016)

Bagheri & Bonadonna[16] derived an expression for the drag coefficient of non-spherical particles:

$$C_d = \frac{24 \cdot k_S}{Re}\left(1 + 0.125\left(\frac{Re \cdot k_N}{k_S}\right)^{2/3}\right) + \frac{0.46 \cdot k_N}{1 + 5330 \cdot \frac{k_S}{Re \cdot k_N}} \tag{S20}$$

with k_N and k_s calculated as follows:

$$k_S = \frac{1}{2}\left(F_S{}^{1/3} + F_S{}^{-1/3}\right) \tag{S21}$$

$$k_N = 10^{\alpha(-logF_N)^{\beta}} \tag{S22}$$

and parameters α and β given as:

$$\alpha = 0.45 + \frac{10}{\exp(2.5 \cdot \log\left({}^{\rho_p}/_{\rho_f}\right) + 30)} \tag{S23}$$

$$\beta = 1 - \frac{37}{\exp(3 \cdot \log\left({}^{\rho_p}/_{\rho_f}\right) + 100)} \tag{S24}$$

Elongation e and flatness f are used as main shape descriptors for calculating F_S and F_N:

$$F_N = f^2 \cdot e\left(\frac{d_{eq}{}^3}{d_l d_i d_s}\right) \tag{S25}$$

$$F_S = f \cdot e^{1.3}\left(\frac{d_{eq}{}^3}{d_l d_i d_s}\right) \tag{S26}$$

Regarding MP fibers, $d_l d_i d_s$ is consequently given as D^2L (also see section S3). d_{eq} is calculated as described in the main publication, which refers to an approximation method that was originally proposed by Bagheri et al.[9] in a previous paper. Finally, w_s can be calculated implicitly after inserting C_d into Equation 1 of the main publication.

S4.8 Dioguardi et al. (2018)

Dioguardi et al.[11] proposed a general expression for the drag coefficient of irregularly-shaped particles, that was deduced from data over a wide range of Reynolds numbers (0.03 ≤ Re ≤ 10,000):

$$C_d = \frac{24}{Re} \cdot \left(\frac{1 - \Psi}{Re} + 1\right)^{0.25} + \frac{24}{Re}(0.1806 \cdot Re^{0.6459})\Psi^{-Re^{0.08}} + \frac{0.4251}{1 + \frac{6.880.95}{Re}\Psi^{5.05}} \tag{S27}$$

Particle shape is characterized by the shape factor Ψ, which is defined as the ratio between the sphericity ϕ and the circularity X (cf. Table S1). C_d is inserted into Equation 1 of the main publication to implicitly calculate w_s.

S4.9 Komar (1980)

Komar[17] studied particles settling at low Reynolds numbers and derived separate formulas for the drag coefficient of ellipsoidal particles and cylinders, respectively.

The expression for ellipsoids is valid for Re < 1 and is basically a correction of Stokes law' based on the Janke shape factor E (see Table S1). Here, it was implemented to describe the settling of MP fragments:

$$C_{d,MP\ fragments} = 24/Re \cdot E^{-0.380} \quad \text{(S28)}$$

Inserting Equation S28 into Equation 1 of the main publication, gives an explicit formula for w_s:

$$w_{s,MP\ fragments} = \frac{1}{18}\, {d_{eq}}^2 \cdot g \cdot \nu^{-1} \left(\frac{\rho_p}{\rho_f} - 1\right) \cdot E^{0.380} \quad \text{(S29)}$$

With respect to MP fibers, Komar's equation for the drag coefficient of cylinders was applied, which is stated to be valid for Re < 2:

$$C_{d,MP\ fibers} = 16.88/Re \cdot (L/D)^{1.664} \quad \text{(S30)}$$

Notably, d_{eq} is defined as cylinder length L in this context – both for computing Re as well as for explicitly calculating w_s via inserting Equation S30 into Equation 1 of the main publication:

$$w_{s,MP\ fibers} = 0.0790\, L^2 \cdot g \cdot \nu^{-1} \left(\frac{\rho_p}{\rho_f} - 1\right) \cdot \left(\frac{L}{D}\right)^{-1.664} \quad \text{(S31)}$$

S4.10 Su et al. (2022)

Su et al.[18] conceptualize a variety of particle shapes as super-ellipsoidal particles, which are determined by their shape parameter ε, elongation e and flatness f. ε can take values between 0 (cubic) and 2 (octahedral) and is set to 1.0 or 0.6 in order to represent MP fragments or MP fibers, respectively (also see Figure S21 taken from the original publication of Su et al.[18]).

Further considered are the dimensionless diameter d_* (computed from d_{eq}, see Equation S11) and the dimensionless velocity w_*, which is defined by:

$$w_* = w_s \cdot \left(\frac{\rho_f}{(\rho_p - \rho_f) \cdot \nu \cdot g}\right)^{1/3} \quad \text{(S32)}$$

Su et al.[18] proposed an expression for explicitly calculating w_* from d_*, ε, e and f:

$$w_* = e^{C_1} f^{C_2} \left(C_3\varepsilon^2 + C_4\varepsilon + 1 - C_3 - C_4\right) \left(\frac{18}{{d_*}^2} + \frac{2.412}{4\sqrt{d_*}}\right)^{-1} \quad \text{(S33)}$$

with C_1, C_2, C_3 and C_4 defined as functions of d_* again:

$$C_1 = 0.11\, log d_* + 0.17$$

$$C_2 = 0.21\, log d_* + 0.17$$

$$C_3 = -0.48\, log d_* + 0.56$$

$$C_4 = 1.05\, log d_* - 0.38$$

Subsequently, w_s can be obtained from w_* via rearranging Equation S32.

S5 Inclination angle of fibers

The inclination of fibers – conceived in the following as the angle (α) between its central axis and the orthogonal plane in direction of gravity – is computed as exemplified in Figure S13. As shown, the height (H) was derived from the detected particle contour. Diameter (d) and length (L) were uniformly assumed for each polymer type or length fraction, respectively, and were determined from microscope images beforehand (detailed in the main publication). Thus, the computation is only an estimate. Moreover, it presupposes a non-curved fiber. This assumption appears to be valid for the detected particles. Only few 600 µm PA 6.6 fibers exhibit a very slight curvature, which is still tolerable. Figure S14 depicts the measured settling velocity and the average computed inclination angle for each detected fiber.

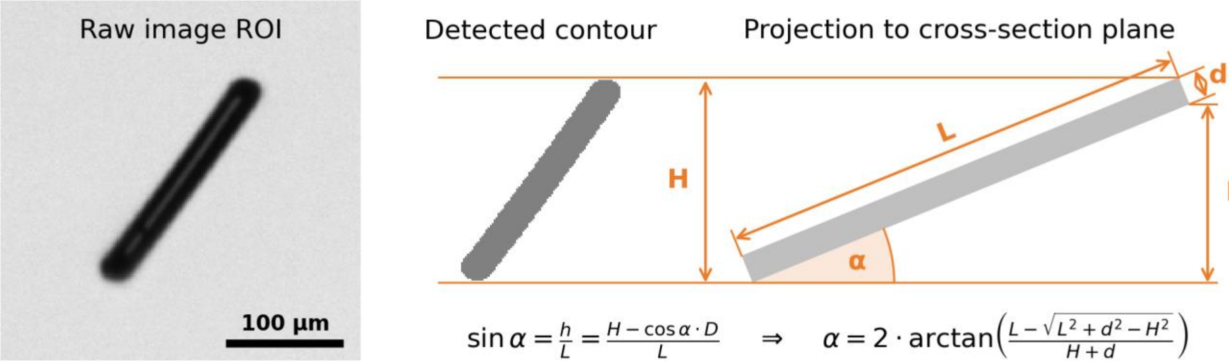

Figure S13. Computation of the inclination angle α of an observed fiber of known length L.

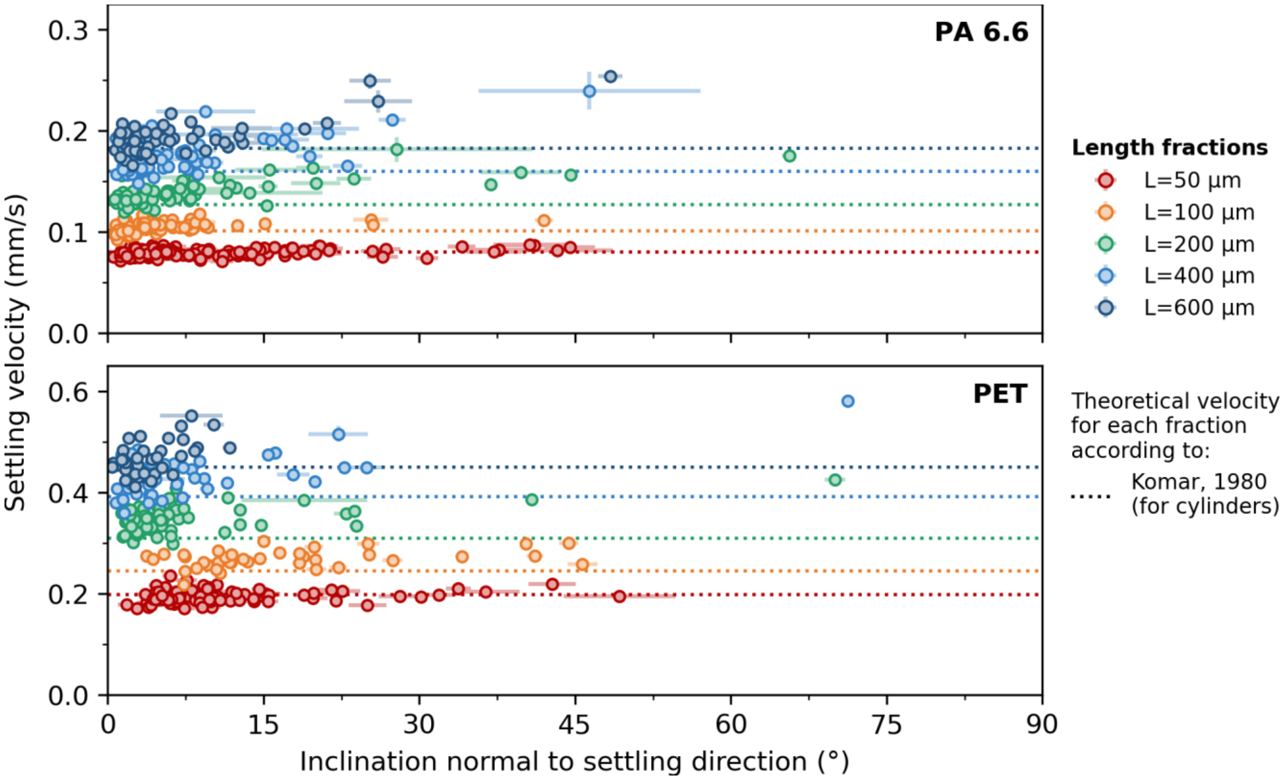

Figure S14. Measured settling velocities versus inclination angles of all investigated fibers. Theoretical velocities calculated for cylinders according to Komar[17] are included for both polymers and all length fractions, respectively.

S6 Measured drag coefficients

The drag coefficient c_d can be computed individually for each observed particle from its measured settling velocity w_s and equivalent diameter d_{eq} by rearranging Equation 1 given in the main text as:

$$c_d = \frac{4 \cdot d_{eq} \cdot g}{3 \cdot {w_s}^2} \left(\frac{\rho_p}{\rho_f} - 1 \right) \tag{S34}$$

Figure S15 depicts the distribution of all measured drag coefficients, while Figure S16 compares the measured drag coefficients with respective Reynolds numbers (cf. Equation 2 of the main text) with Stokes' law providing a reasonable lower estimate as was discussed in the main text. A table of all measured drag coefficients and Reynolds numbers is provided in the associated Zenodo repository[3] as a file named *'all_drag_coefficients.csv'*.

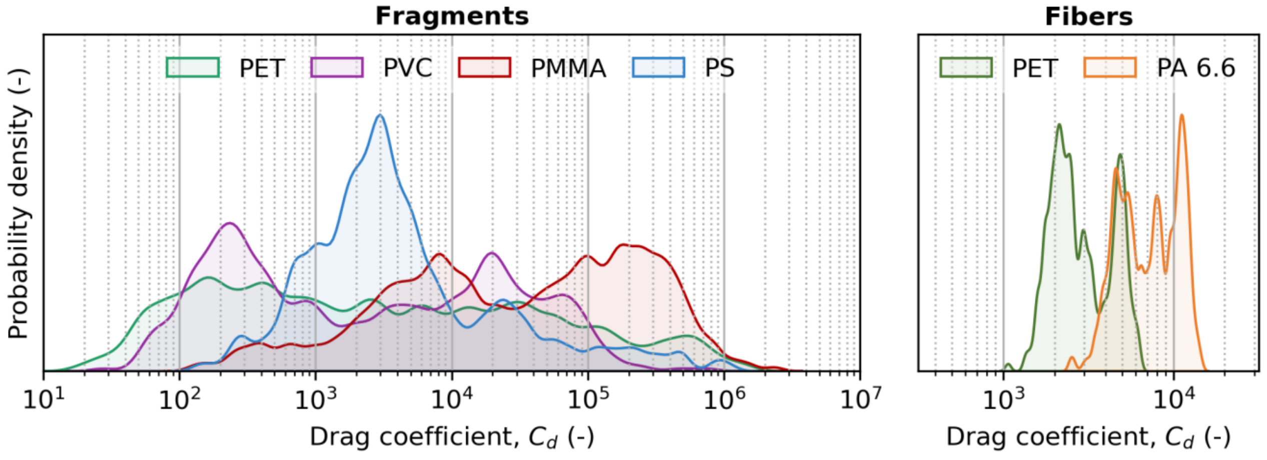

Figure S15. Distribution of drag coefficients as measured for MP fragments (left-hand side) and fibers (right-hand side). Density plots created via log-transformed kernel density estimation.

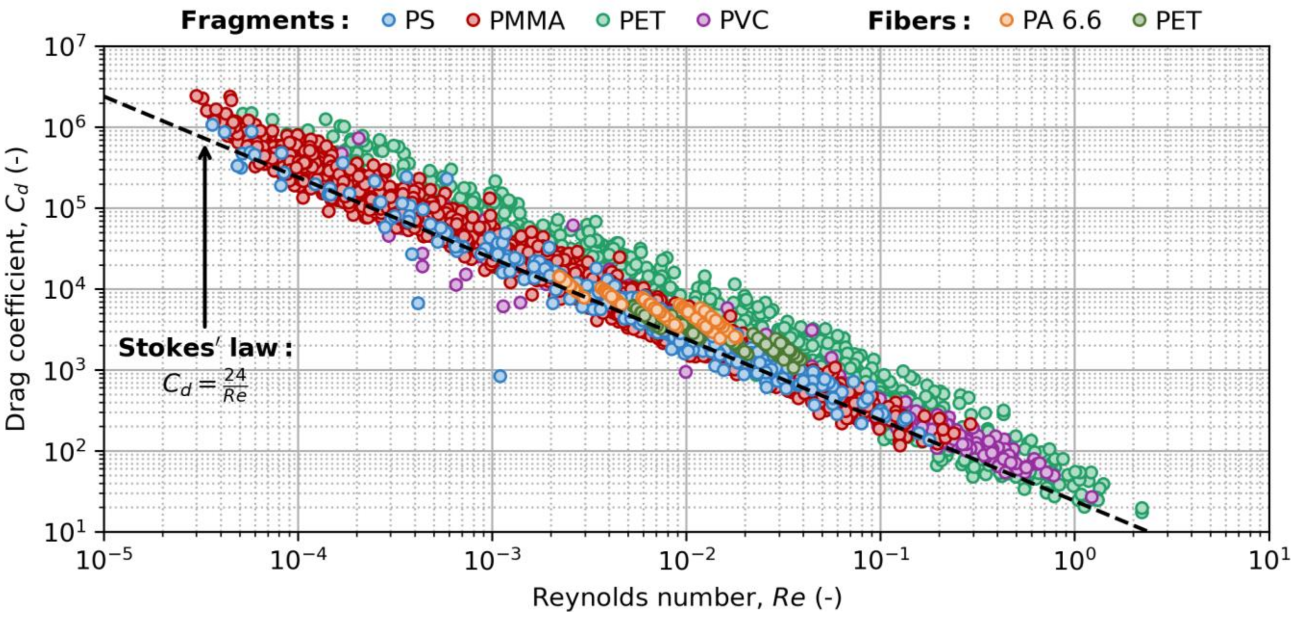

Figure S16. Drag coefficient versus Reynolds number as computed for all measured MP fragments and fibers.

S7 Comparison of terminal settling velocity models

Ten different models for computing a particle's terminal velocity were tested based on the empirical data acquired for small MP fragments and fibers. The computation of necessary size and shape descriptors is detailed in section S3, whereas the implementation of each model is described in section S4. In the following, the models are additionally numbered according to the subsections of S4 (Model 1–10). As detailed in the main publication (cf. section 'Terminal settling velocity models'), the performance of each model is assessed by means of the logarithmic coefficient of determination (R^2_{log}), the coefficient of determination (R^2) and the average absolute relative error (|AE|).

Four different definitions of the equivalent diameter were tested for each model (excluding for the computation of C_d and Re itself, if a respective diameter was decidedly specified by the authors). This was done as the characterization of particle size and shape from images is prone to error for irregular particles (see respective discussions in the main manuscript). Thus, the optimal definition of the equivalent diameter might deviate from the one, which was originally specified for the respective model. The tested diameters were collected from all models and are annotated and computed as follows for MP fragments:

$$d_{eq} = d_{eq,Bagheri} \text{ (see Equation S3)}, \; d_1 = \sqrt[3]{d_l d_i d_s} \;, \; d_2 = \sqrt[3]{d_l {d_s}^2} \text{ and } d_3 = \sqrt{4 d_l d_i / \pi}$$

Consequently, the respective diameters for MP fibers, described as cylinders with diameter D and length L, are defined as:

$$d_{eq} = \sqrt{1.5 D^2 L}, \; d_1 = \sqrt[3]{D^2 L} \;, \; d_2 = d_1 \text{ and } d_3 = \sqrt{4DL/\pi}$$

The original specification of d for each model is indicated by the gray background in Table S2–Table S4 (with d_{eq} as default setting). Waldschläger & Schüttrumpf[5] (Model 1, see S4.1), Khatmullia & Isachenko[15] (Model 6, see S4.6) and Komar[17] (Model 9, see S4.9) proposed specific models for settling fibers based on their actual diameters D. When considering MP fibers and these models, d was thus not varied but always set as D following the respective authors (also indicated in Table S2–Table S4).

Figure S17 (Model 1–5) and Figure S18 (Model 6–10) compare the predictions of each model with the respectively measured settling velocities. As Goral et al.[7], Dioguardi et al.[11] and Komar[17] specified Reynolds number limits for their models (cf. S4.2, S4.8 and S4.9), Figure S19 similarly shows predictions versus measurements for those three models, but excludes all particles that do not meet the respective criteria for Re.

The figures only display R^2_{log}, R^2 and |AE| as calculated for the entire data, that is respectively shown. The subsequent tables comprise the performance measures as computed for all data, MP fragments and MP fibers (Table S2) and for each of the investigated particle samples, respectively: PS fragments, PMMA fragments and PET fragments (Table S3) as well as PVC fragments, PA 6.6 fibers and PET fibers (Table S4).

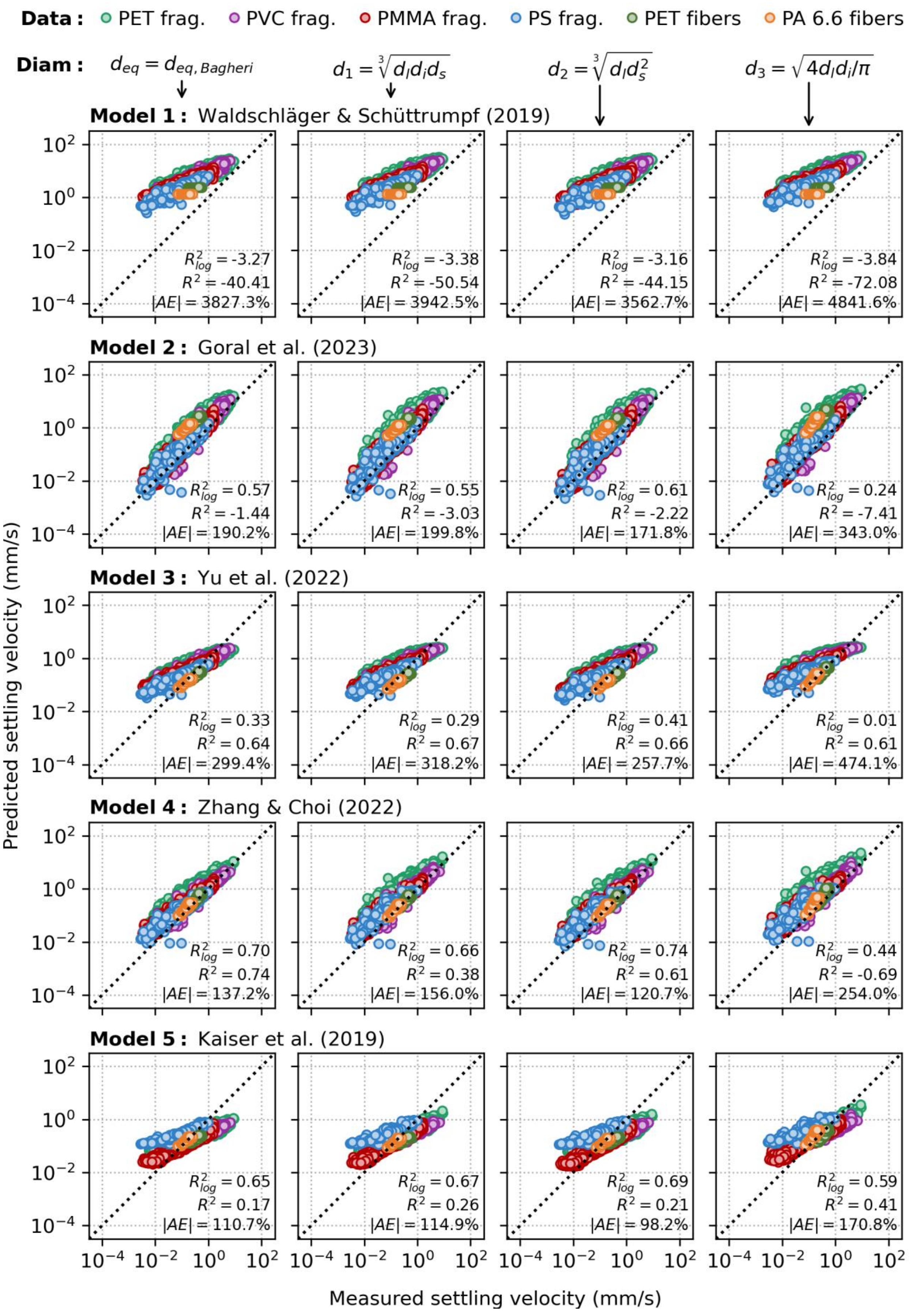

Figure S17. Measured versus predicted settling velocities of different models (rows, Models 1–5) and for various diameter approximations as input (columns).

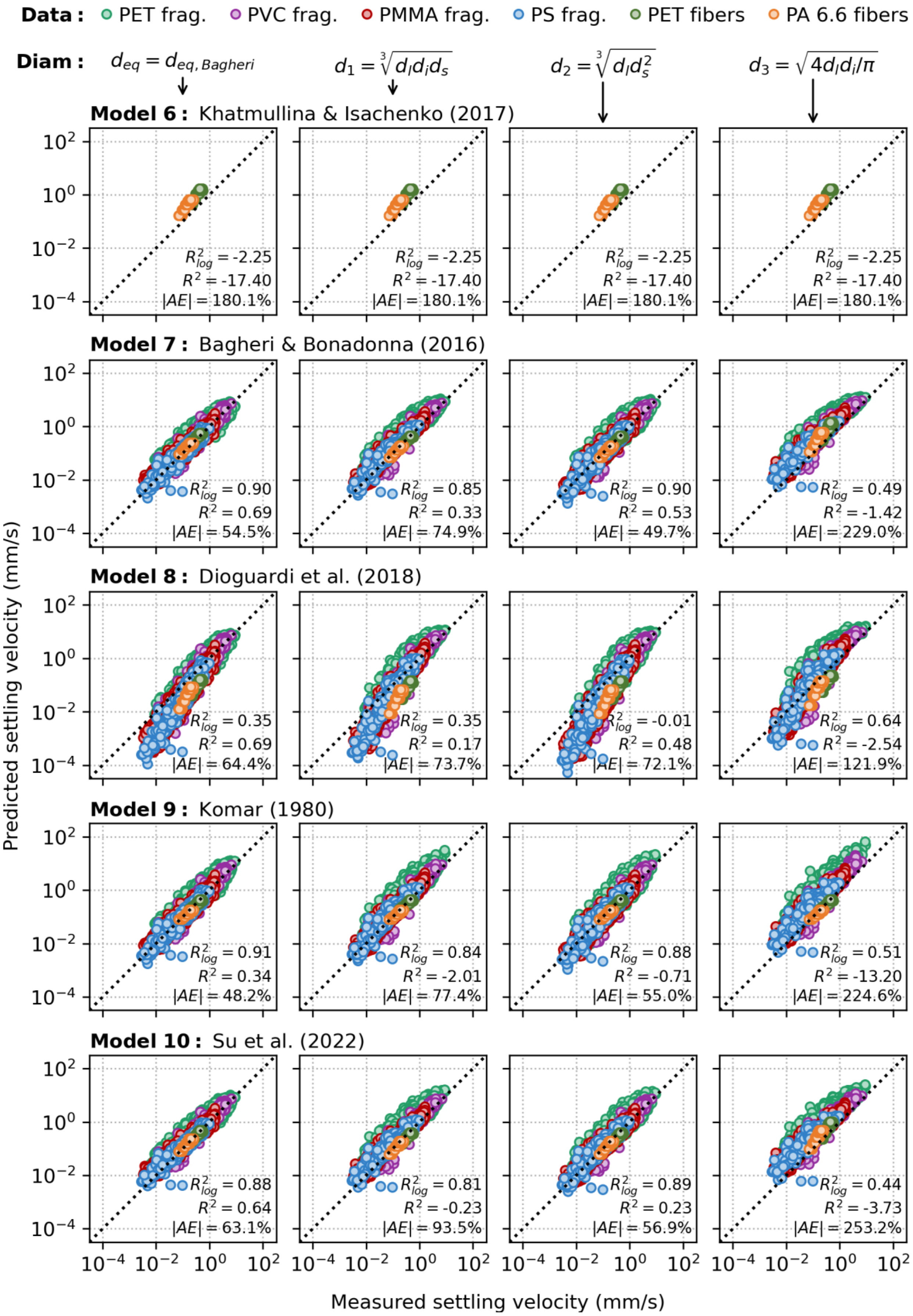

Figure S18. Measured versus predicted settling velocities of different models (rows, Models 6–10) and for various diameter approximations as input (columns).

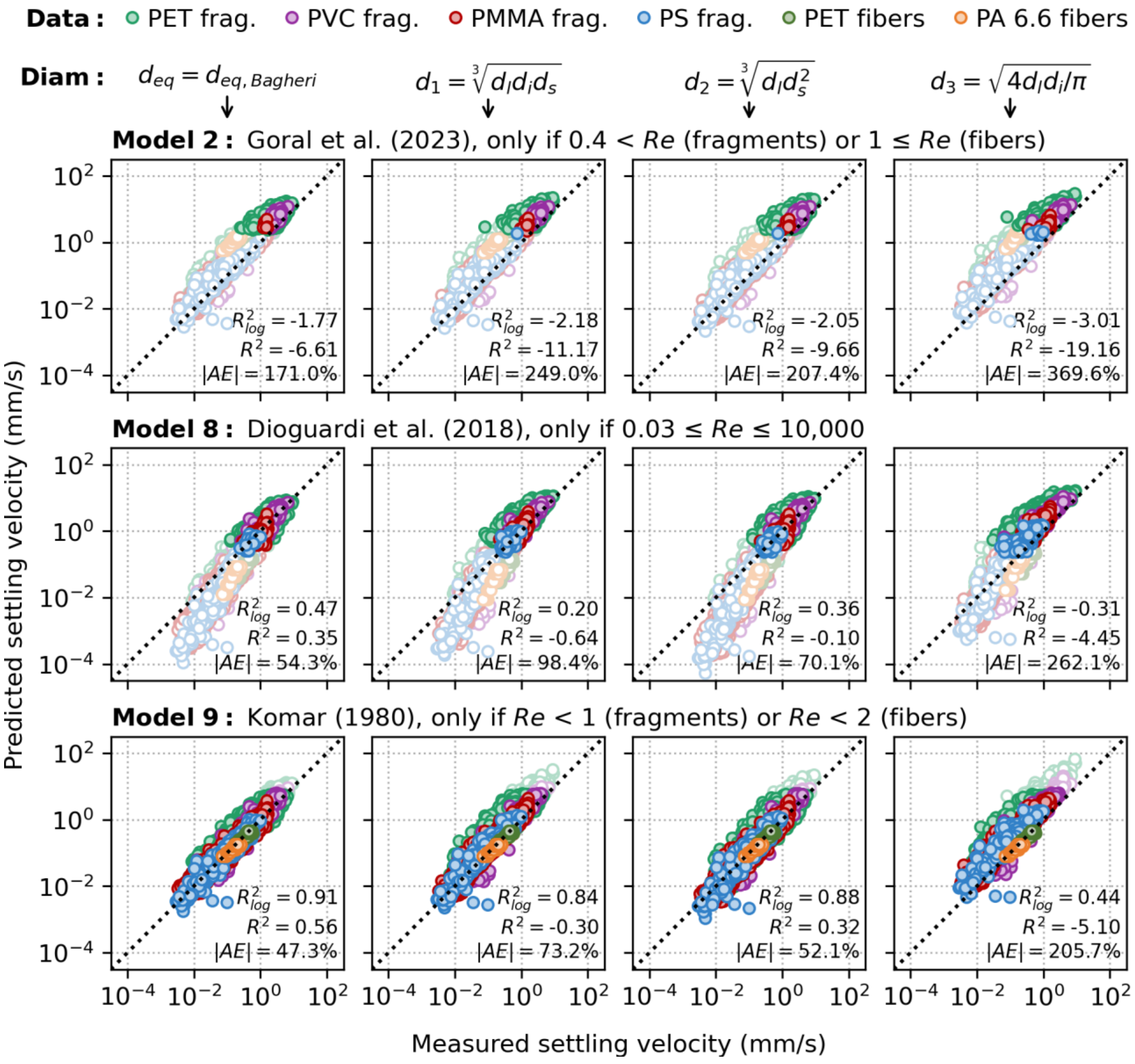

Figure S19. Measured versus predicted settling velocities for all models with specified Reynolds number limits (rows, Model 2, 8 and 9) and for various diameter estimates as input (columns). All data, that does not meet respective Re criteria is depicted pale and excluded from the calculation of the given performance measures.

Table S2. Model performances for all samples, MP fragments and fibers, respectively. Re limits for M2, M8 and M9 were both applied (annotated, see footnote) as well as not implemented.

Model	Diam.	All samples R^2_{log}	All samples R^2	All samples |AE| (%)	MP fragments R^2_{log}	MP fragments R^2	MP fragments |AE| (%)	MP fibers R^2_{log}	MP fibers R^2	MP fibers |AE| (%)
**M1** Waldschl. & Schüttr.	d_{eq}	-3.27	-40.41	3827.3	-3.08	-40.42	4402.2	-15.03	-176.29	923.0
	d_1	-3.38	-50.54	3942.5	-3.19	-50.71	4540.2	unequivocal definition of fiber size and shape in M1		
	d_2	-3.16	-44.15	3562.7	-2.96	-44.23	4085.1			
	d_3	-3.84	-72.08	4841.6	-3.66	-72.61	5617.1			
**M2** Goral et al.	d_{eq}	0.57	-1.44	190.2	0.79	-1.10	116.0	-9.96	-100.12	565.2
	d_1	0.55	-3.03	199.8	0.73	-2.84	146.3	-8.24	-68.49	470.0
	d_2	0.61	-2.22	171.8	0.79	-2.02	112.8	equivalent to d_1		
	d_3	0.24	-7.41	343.0	0.54	-6.44	244.5	-14.20	-295.60	840.7
M2* Goral et al.	d_{eq}	-1.77	-6.61	171.0	-1.77	-6.61	171.0	no data within Reynolds number limits (cf. below)		
	d_1	-2.18	-11.17	249.0	-2.18	-11.17	249.0			
	d_2	-2.05	-9.66	207.4	-2.05	-9.66	207.4			
	d_3	-3.01	-19.16	369.6	-3.01	-19.16	369.6			
**M3** Yu et al.	d_{eq}	0.33	0.64	299.4	0.31	0.63	355.5	0.81	0.65	15.9
	d_1	0.29	0.67	318.2	0.27	0.67	376.4	0.64	0.46	23.9
	d_2	0.41	0.66	257.7	0.40	0.66	304.0	equivalent to d_1		
	d_3	0.01	0.61	474.1	-0.02	0.61	564.0	0.86	0.86	19.8
**M4** Zhang & Choi	d_{eq}	0.70	0.74	137.2	0.70	0.74	158.3	0.74	0.52	30.5
	d_1	0.66	0.38	156.0	0.65	0.37	183.3	0.88	0.80	17.6
	d_2	0.74	0.61	120.7	0.74	0.60	141.1	equivalent to d_1		
	d_3	0.44	-0.69	254.0	0.44	-0.71	290.3	0.05	-2.38	70.3
**M5** Kaiser et al.	d_{eq}	0.65	0.17	110.7	0.65	0.16	126.7	0.51	0.28	30.0
	d_1	0.67	0.26	114.9	0.68	0.25	132.6	equivalent to d_2		
	d_2	0.69	0.21	98.2	0.69	0.20	112.6	0.39	0.12	25.3
	d_3	0.59	0.41	170.8	0.59	0.40	195.1	0.41	0.30	47.6
**M6** Khatm. & Isach.	d_{eq}	-2.25	-17.40	180.1	M6 only established for MP fibers			-2.25	-17.40	180.1
	d_1	unequivocal definition of fiber size and shape in M6						unequivocal definition of fiber size and shape in M6		
	d_2									
	d_3									
**M7** Bagheri & Bonad.	d_{eq}	0.90	0.69	54.5	0.90	0.69	61.1	0.87	0.80	21.5
	d_1	0.85	0.33	74.9	0.85	0.32	88.1	0.97	0.96	8.3
	d_2	0.90	0.53	49.7	0.90	0.53	57.8	equivalent to d_1		
	d_3	0.49	-1.42	229.0	0.52	-1.42	250.8	-0.95	-9.18	118.9
**M8** Dioguardi et al.	d_{eq}	0.35	0.69	64.4	0.45	0.70	62.9	-4.79	-0.91	72.2
	d_1	0.35	0.17	73.7	0.51	0.17	72.7	-7.59	-1.23	78.7
	d_2	-0.01	0.48	72.1	0.14	0.48	70.8	equivalent to d_1		
	d_3	0.64	-2.54	121.9	0.70	-2.60	134.8	-2.13	-0.19	56.4
M8† Dioguardi et al.	d_{eq}	0.47	0.35	54.3	0.47	0.35	54.3	no data within Reynolds number limits (cf. below)		
	d_1	0.20	-0.64	98.4	0.20	-0.64	98.4			
	d_2	0.36	-0.10	70.1	0.36	-0.10	70.1			
	d_3	-0.31	-4.45	262.1	-0.31	-4.45	262.1			
**M9** Komar	d_{eq}	0.91	0.34	48.2	0.91	0.33	56.4	0.97	0.95	6.9
	d_1	0.84	-2.01	77.4	0.84	-2.06	91.4	unequivocal definition of fiber size and shape in M9		
	d_2	0.88	-0.71	55.0	0.87	-0.74	64.5			
	d_3	0.51	-13.20	224.6	0.49	-13.43	267.6			
M9‡ Komar	d_{eq}	0.91	0.56	47.3	0.90	0.55	55.4	0.97	0.95	6.9
	d_1	0.84	-0.30	73.2	0.83	-0.32	86.7	unequivocal definition of fiber size and shape in M9		
	d_2	0.88	0.32	52.1	0.87	0.31	61.3			
	d_3	0.44	-5.10	205.7	0.40	-5.22	248.6			
**M10** Su et al.	d_{eq}	0.88	0.64	63.1	0.88	0.64	73.8	0.96	0.96	9.3
	d_1	0.81	-0.23	93.5	0.81	-0.25	108.7	0.87	0.85	16.4
	d_2	0.89	0.23	56.9	0.89	0.22	64.9	equivalent to d_1		
	d_3	0.44	-3.73	253.2	0.44	-3.79	289.3	0.01	-2.46	70.6

Applied limits for Re: * Fragments: $0.4 < Re$; Fibers: $1 \le Re$, † $0.03 \le Re \le 10{,}000$; ‡ Fragments: $Re < 1$; Fibers: $Re < 2$

Table S3. Model performances for all samples, PS, PMMA and PET fragments, respectively. Re limits for M2, M8 and M9 were both applied (annotated, see footnote) as well as not implemented.

Model	Diam.	PS fragments			PMMA fragments			PET fragments		
		R^2_{log}	R^2	|AE| (%)	R^2_{log}	R^2	|AE| (%)	R^2_{log}	R^2	|AE| (%)
**M1** Waldschl. & Schüttr.	d_{eq}	-7.21	-345.61	2661.1	-6.86	-176.71	5771.8	-2.17	-38.71	3277.4
	d_1	-7.69	-418.04	2910.9	-6.96	-201.96	5827.4	-2.38	-52.28	3617.9
	d_2	-7.27	-374.03	2657.9	-6.54	-175.24	5238.2	-2.13	-44.58	3170.4
	d_3	-8.67	-571.46	3543.8	-7.82	-286.85	7210.3	-2.87	-77.17	4623.8
**M2** Goral et al.	d_{eq}	0.67	-0.88	74.8	0.83	-0.05	68.5	0.49	-2.01	254.4
	d_1	0.51	-2.14	105.5	0.79	-0.57	79.7	0.34	-4.81	336.9
	d_2	0.61	-1.56	84.5	0.85	-0.23	58.3	0.49	-3.53	259.4
	d_3	0.15	-5.29	173.1	0.56	-2.35	149.1	-0.02	-10.26	540.5
M2* Goral et al.	d_{eq}	no data within Re limits			-70.20	-216.92	133.3	-1.56	-6.06	193.0
	d_1	0.00	0.00	169.2	-25.86	-94.77	150.2	-2.05	-10.89	288.6
	d_2	0.00	0.00	160.5	-26.15	-95.50	143.7	-1.88	-9.37	238.3
	d_3	-14.03	-47.54	208.5	-8.61	-57.48	218.2	-3.00	-19.19	436.4
**M3** Yu et al.	d_{eq}	0.23	0.39	166.5	-0.46	0.43	473.5	0.43	0.48	298.0
	d_1	0.03	-0.03	205.1	-0.50	0.33	482.7	0.35	0.53	349.9
	d_2	0.23	0.27	164.8	-0.24	0.51	390.9	0.48	0.51	272.0
	d_3	-0.49	-1.48	314.8	-1.10	-0.56	719.1	0.08	0.47	543.7
**M4** Zhang & Choi	d_{eq}	0.66	0.67	80.5	0.53	0.79	155.5	0.49	0.57	260.4
	d_1	0.51	0.25	108.9	0.49	0.65	165.8	0.37	-0.05	327.2
	d_2	0.64	0.52	84.3	0.63	0.78	127.4	0.51	0.34	250.3
	d_3	0.16	-1.01	175.2	0.15	-0.04	262.2	0.05	-1.79	516.7
**M5** Kaiser et al.	d_{eq}	-0.40	-0.47	323.4	0.66	0.48	103.7	0.51	-0.09	112.1
	d_1	-0.63	-1.49	368.5	0.68	0.57	103.3	0.59	0.05	118.6
	d_2	-0.43	-0.79	325.5	0.75	0.50	79.3	0.55	-0.03	106.2
	d_3	-1.19	-5.05	496.6	0.43	0.72	177.9	0.61	0.28	160.6
**M6** Khatm. & Isach.	d_{eq}	M6 only established for MP fibers			M6 only established for MP fibers			M6 only established for MP fibers		
	d_1									
	d_2									
	d_3									
**M7** Bagheri & Bonad.	d_{eq}	0.87	0.77	30.4	0.90	0.78	44.3	0.76	0.53	126.3
	d_1	0.75	0.25	59.9	0.88	0.58	53.6	0.61	-0.03	199.2
	d_2	0.81	0.56	40.3	0.91	0.73	36.5	0.76	0.27	125.9
	d_3	0.13	-3.17	176.9	0.39	-1.35	194.6	0.07	-2.43	481.3
**M8** Dioguardi et al.	d_{eq}	-0.48	0.55	58.8	-0.24	0.69	68.1	0.79	0.55	65.0
	d_1	0.15	0.68	44.3	-0.08	0.71	63.8	0.73	-0.30	118.7
	d_2	-0.58	0.59	54.4	-1.03	0.68	74.1	0.63	0.19	84.7
	d_3	0.55	-1.21	69.6	0.75	-0.74	52.4	0.27	-4.40	371.3
***M8*†** Dioguardi et al.	d_{eq}	0.22	0.30	22.7	-0.20	-0.29	29.1	0.38	0.25	74.4
	d_1	-0.01	-0.15	30.1	0.26	-0.44	35.0	-0.05	-0.98	150.4
	d_2	0.16	0.19	24.1	0.04	-0.32	32.3	0.19	-0.32	101.8
	d_3	-0.86	-3.39	108.5	-0.55	-4.22	107.8	-0.71	-5.59	414.7
**M9** Komar	d_{eq}	0.86	0.74	29.7	0.93	0.73	35.0	0.76	-0.03	126.3
	d_1	0.73	-0.01	60.1	0.90	0.39	44.6	0.55	-4.01	228.7
	d_2	0.78	0.41	42.6	0.88	0.63	39.0	0.72	-1.85	144.5
	d_3	0.10	-4.98	182.2	0.49	-2.72	167.0	-0.13	-22.33	599.5
***M9*‡** Komar	d_{eq}	0.86	0.74	29.7	0.93	0.73	35.0	0.74	0.31	125.9
	d_1	0.73	-0.01	60.1	0.90	0.39	44.6	0.51	-1.29	224.4
	d_2	0.78	0.41	42.6	0.88	0.63	39.0	0.71	-0.11	136.8
	d_3	0.10	-4.98	182.2	0.49	-2.28	166.8	-0.58	-13.11	626.2
**M10** Su et al.	d_{eq}	0.87	0.78	33.3	0.87	0.79	58.0	0.71	0.44	148.3
	d_1	0.69	0.12	71.9	0.83	0.54	70.8	0.52	-0.99	241.8
	d_2	0.81	0.50	43.8	0.92	0.73	37.9	0.71	-0.26	150.5
	d_3	0.02	-3.94	198.8	0.29	-1.71	222.0	-0.07	-6.35	570.1

Applied limits for Re: * Fragments: $0.4 < Re$; Fibers: $1 \leq Re$, † $0.03 \leq Re \leq 10{,}000$; ‡Fragments: $Re < 1$; Fibers: $Re < 2$

Table S4. Model performances for PVC fragments, PA 6.6 fibers and PET fibers, respectively. Re limits for M2, M8 and M9 were both applied (annotated, see footnote) as well as not implemented.

Model	Diam.	PVC fragments			PA 6.6 fibers			PET fibers		
		R^2_{log}	R^2	|AE| (%)	R^2_{log}	R^2	|AE| (%)	R^2_{log}	R^2	|AE| (%)
**M1** Waldschl. & Schüttr.	d_{eq}	-2.91	-65.16	2703.1	-46.40	-701.73	1059.4	-36.41	-385.61	734.8
	d_1	-2.96	-74.08	2713.5	unequivocal definition of fiber size and shape in M1			unequivocal definition of fiber size and shape in M1		
	d_2	-2.84	-67.70	2572.6						
	d_3	-3.31	-99.58	3151.1						
**M2** Goral et al.	d_{eq}	0.83	-0.98	84.2	-31.19	-368.56	633.7	-24.88	-227.39	470.7
	d_1	0.80	-1.78	94.8	-26.40	-256.34	528.7	-20.50	-155.09	389.0
	d_2	0.83	-1.28	82.4	equivalent to d_1			equivalent to d_1		
	d_3	0.67	-4.32	142.0	-42.78	-1049.5	932.8	-36.18	-677.36	713.6
M2* Goral et al.	d_{eq}	-5.23	-11.88	111.7	no data within Reynolds number limits (cf. below)			no data within Reynolds number limits (cf. below)		
	d_1	-6.51	-16.00	135.4						
	d_2	-6.01	-13.99	122.6						
	d_3	-7.52	-26.63	186.9						
**M3** Yu et al.	d_{eq}	0.57	0.70	171.9	0.94	0.91	5.8	-0.17	-0.10	29.8
	d_1	0.58	0.76	170.0	0.76	0.71	13.8	-1.03	-0.66	37.8
	d_2	0.62	0.73	151.8	equivalent to d_1			equivalent to d_1		
	d_3	0.43	0.77	231.6	0.49	-0.17	25.6	0.81	0.85	11.9
**M4** Zhang & Choi	d_{eq}	0.90	0.93	55.5	0.10	-1.13	37.3	0.60	0.01	21.0
	d_1	0.89	0.83	60.8	0.57	-0.01	22.5	0.84	0.62	10.9
	d_2	0.91	0.90	49.7	equivalent to d_1			equivalent to d_1		
	d_3	0.80	0.44	97.5	-1.94	-10.59	78.7	-1.06	-6.81	58.6
**M5** Kaiser et al.	d_{eq}	0.47	-0.12	87.7	0.67	0.63	21.1	-1.79	-1.18	42.4
	d_1	0.53	-0.05	86.0	equivalent to d_2			equivalent to d_2		
	d_2	0.50	-0.10	84.2	0.92	0.92	8.7	-2.95	-1.77	48.2
	d_3	0.62	0.11	91.4	-1.12	-5.50	64.4	0.17	0.41	24.4
**M6** Khatm. & Isach.	d_{eq}	M6 only established for MP fibers			-7.43	-40.37	177.1	-8.31	-47.09	184.2
	d_1				unequivocal definition of fiber size and shape in M1			unequivocal definition of fiber size and shape in M6		
	d_2									
	d_3									
**M7** Bagheri & Bonad.	d_{eq}	0.94	0.77	34.5	0.63	0.39	22.5	0.68	0.51	20.1
	d_1	0.92	0.51	45.9	0.91	0.91	8.0	0.90	0.89	8.6
	d_2	0.94	0.69	33.1	equivalent to d_1			equivalent to d_1		
	d_3	0.74	-1.17	120.1	-4.32	-26.96	121.0	-4.23	-24.32	115.9
**M8** Dioguardi et al.	d_{eq}	0.66	0.79	44.1	-14.88	-3.00	71.7	-14.34	-4.05	72.8
	d_1	0.66	0.51	48.5	-23.20	-3.99	79.0	-20.80	-4.84	78.4
	d_2	0.51	0.71	48.2	equivalent to d_1			equivalent to d_1		
	d_3	0.77	-1.68	80.8	-7.31	-0.83	53.1	-7.69	-2.32	61.0
M8† Dioguardi et al.	d_{eq}	0.58	0.45	28.9	no data within Reynolds number limits (cf. below)			no data within Reynolds number limits (cf. below)		
	d_1	0.50	-0.21	39.6						
	d_2	0.55	0.25	31.1						
	d_3	-0.31	-4.65	119.3						
**M9** Komar	d_{eq}	0.94	0.55	35.6	0.93	0.90	6.1	0.91	0.87	8.1
	d_1	0.90	-0.24	51.4	unequivocal definition of fiber size and shape in M9			unequivocal definition of fiber size and shape in M9		
	d_2	0.92	0.31	37.9						
	d_3	0.70	-5.57	134.4						
M9‡ Komar	d_{eq}	0.94	0.60	34.9	0.93	0.90	6.1	0.91	0.87	8.1
	d_1	0.90	0.04	48.7	unequivocal definition of fiber size and shape in M9			unequivocal definition of fiber size and shape in M9		
	d_2	0.92	0.41	36.8						
	d_3	0.69	-2.77	122.1						
**M10** Su et al.	d_{eq}	0.94	0.77	35.5	0.86	0.71	11.5	0.95	0.93	6.2
	d_1	0.91	0.39	48.6	0.76	0.84	13.3	0.46	0.56	20.8
	d_2	0.94	0.65	33.5	equivalent to d_1			equivalent to d_1		
	d_3	0.72	-2.04	126.9	-2.03	-11.80	78.3	-1.15	-6.79	60.0

Applied limits for Re: * Fragments: 0.4 < Re; Fibers: 1 ≤ Re, † 0.03 ≤ Re ≤ 10,000; ‡Fragments: Re < 1; Fibers: Re < 2

S8 Testing the model of Su et al.[18] for large microplastics

The terminal settling velocity model of Su et al.[18] appears to be suitable for predicting the settling velocities of small MP fragments and fibers tested in this study (see section S7). Yet, in contrast to most of the other tested models, it has not been tested for other data sets on larger MP particles thus far. Figure S20 depicts settling velocities of ten different types of MP particles as measured by Goral et al.[7] versus corresponding predictions according to Su et al.[18]. In order to apply the model, a shape parameter ε had to be set to characterize the shape of a particle within the proposed framework of super-ellipsoids – apart from determining the descriptors elongation e and flatness f. The shape parameter ε can take values between 0.0 (cube) and 2.0 (octahedron) – with 1.0 describing spherical shape. This is illustrated by Figure S21, which was extracted from the original publication of Su et al.[18]. Although the choice of ε is not always straight forward with respect to each possibly considered particle shape (e.g. tetrahedrons), it appears to be a very sensitive parameter with respect to the results for large MP. Figure S20a shows the predictions according to the values for ε that were initially chosen by the authors (ranging from 0.2 to 1.4 for the considered particle shapes) and includes inaccurate predictions for certain particle types – as is indicated by an overall negative coefficient of determination. This changes, if ε is only varied between 0.8 and 1.0 as is depicted in Figure S20b – yet, these choices are not coherent with the original definition of ε.

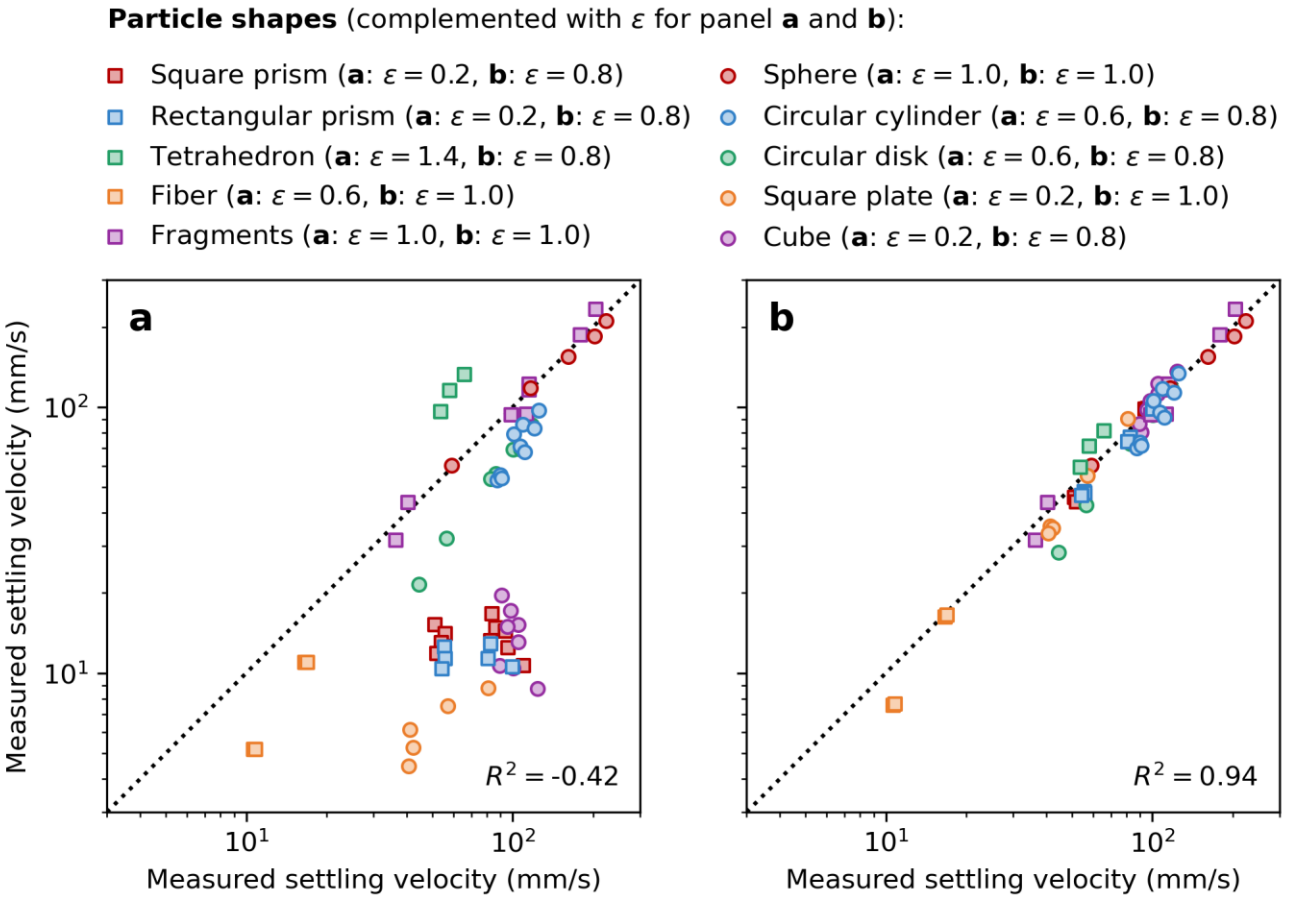

Figure S20. Measured velocities of different types of large MPs (data by Goral et al.[7]) and corresponding predicted velocities according to Su et al.[18]. Initial and alternative choices of shape parameter ε for the considered particle shapes are depicted in panel a and b, respectively, and specified in the figure's legend.

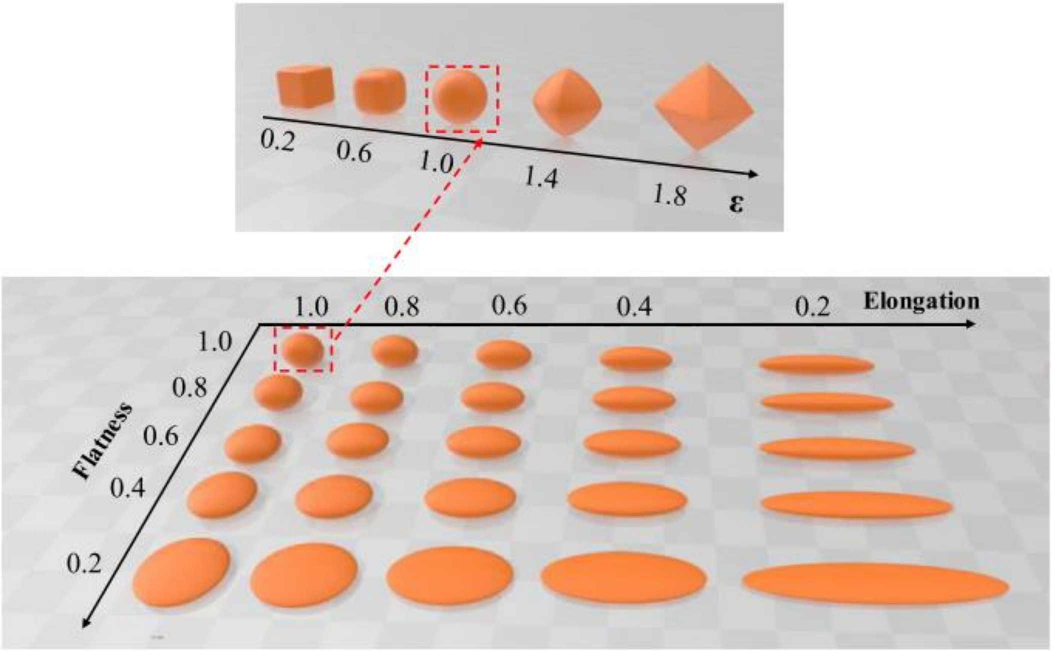

Figure S21. Shapes of super-ellipsoidal particles for varying values of elongation and flatness as well as of the shape parameter ε. Figure originally titled "Illustration of generated super-ellipsoidal particles". Reprinted from Powder Technology, 33 (12), Su, D.; Fan, M.; Han, W.; Chen, X., A terminal-velocity model for super-ellipsoidal particles, 103882, 2022, with permission from Elsevier.

S9 References

1. Dittmar, S.; Ruhl, A. S.; Jekel, M. Optimized and Validated Settling Velocity Measurement for Small Microplastic Particles (10-400 µm). *ACS EST Water* **2023**. DOI: 10.1021/acsestwater.3c00457.
2. Kaskas, A. A. *Berechnung der stationären und instationären Bewegung von Kugeln in ruhenden und strömenden Medien*. Diplomarbeit; TU Berlin (Lehrstuhl fur Thermodynamik und Verfahrenstechnik), 1964.
3. Dittmar, S. Additional data for "Settling Velocities of Small Microplastic Fragments and Fibers" **2023**. DOI: 10.5281/zenodo.10049927.
4. Cole, M. A novel method for preparing microplastic fibers. *Sci. rep.* **2016**, *6*, 34519. DOI: 10.1038/srep34519.
5. Waldschläger, K.; Schüttrumpf, H. Effects of Particle Properties on the Settling and Rise Velocities of Microplastics in Freshwater under Laboratory Conditions. *Environ. Sci. Technol.* **2019**, *53* (4), 1958–1966. DOI: 10.1021/acs.est.8b06794.
6. van Melkebeke, M.; Janssen, C. R.; Meester, S. de. Characteristics and Sinking Behavior of Typical Microplastics including the Potential Effect of Biofouling: Implications for Remediation. *Environ. Sci. Technol.* **2020.** DOI: 10.1021/acs.est.9b07378.
7. Goral, K. D.; Guler, H. G.; Larsen, B. E.; Carstensen, S.; Christensen, E. D.; Kerpen, N. B.; Schlurmann, T.; Fuhrman, D. R. Settling velocity of microplastic particles having regular and irregular shapes. *Environ. Res.* **2023**, *228*, 115783. DOI: 10.1016/j.envres.2023.115783. Published Online: Apr. 5, 2023.
8. Bottema, M. J. Circularity of objects in images. In *2000 IEEE International Conference on Acoustics, Speech, and Signal Processing. Proceedings (Cat. No.00CH37100)*; IEEE, 2000; pp 2247–2250. DOI: 10.1109/ICASSP.2000.859286.
9. Bagheri, G. H.; Bonadonna, C.; Manzella, I.; Vonlanthen, P. On the characterization of size and shape of irregular particles. *Powder Technol.* **2015**, *270*, 141–153. DOI: 10.1016/j.powtec.2014.10.015.
10. Dellino, P.; Mele, D.; Bonasia, R.; Braia, G.; La Volpe, L.; Sulpizio, R. The analysis of the influence of pumice shape on its terminal velocity. *Geophys. Res. Lett.* **2005**, *32* (21). DOI: 10.1029/2005GL023954.
11. Dioguardi, F.; Mele, D.; Dellino, P. A New One-Equation Model of Fluid Drag for Irregularly Shaped Particles Valid Over a Wide Range of Reynolds Number. *J. Geophys. Res. Solid Earth* **2018**, *123* (1), 144–156. DOI: 10.1002/2017JB014926.
12. Yu, Z.; Yang, G.; Zhang, W. A new model for the terminal settling velocity of microplastics. *Mar. Pollut. Bull.* **2022**, *176*, 113449. DOI: 10.1016/j.marpolbul.2022.113449. Published Online: Feb. 17, 2022.
13. Zhang, J.; Choi, C. E. Improved Settling Velocity for Microplastic Fibers: A New Shape-Dependent Drag Model. *Environ. Sci. Technol.* **2022**, *56* (2), 962–973. DOI: 10.1021/acs.est.1c06188. Published Online: Dec. 28, 2021.
14. Kaiser, D.; Estelmann, A.; Kowalski, N.; Glockzin, M.; Waniek, J. J. Sinking velocity of sub-millimeter microplastic. *Mar. Pollut. Bull.* **2019**, *139*, 214–220. DOI: 10.1016/j.marpolbul.2018.12.035.

15. Khatmullina, L.; Isachenko, I. Settling velocity of microplastic particles of regular shapes. *Mar. Pollut. Bull.* **2017**, *114* (2), 871–880. DOI: 10.1016/j.marpolbul.2016.11.024.
16. Bagheri, G.; Bonadonna, C. On the drag of freely falling non-spherical particles. *Powder Technol.* **2016**, *301*, 526–544. DOI: 10.1016/j.powtec.2016.06.015.
17. Komar, P. D. Settling Velocities of Circular Cylinders at Low Reynolds Numbers. *J. Geol.* **1980**, *88* (3), 327–336. DOI: 10.1086/628510.
18. Su, D.; Fan, M.; Han, W.; Chen, X. A terminal-velocity model for super-ellipsoidal particles. *Adv. Powder Technol.* **2022**, *33* (12), 103882. DOI: 10.1016/j.apt.2022.103882.

Figure S21 reprinted from Powder Technology, 33 (12), Su, D.; Fan, M.; Han, W.; Chen, X., A terminal-velocity model for super-ellipsoidal particles, 103882, 2022, with permission from Elsevier.
